# Apolipoprotein O modulates cholesterol metabolism via NRF2/CYB5R3 independent of LDL receptor

**DOI:** 10.1038/s41419-024-06778-4

**Published:** 2024-06-03

**Authors:** Jin Chen, Jiarui Hu, Xin Guo, Yang Yang, Donglu Qin, Xiaoyu Tang, Zhijie Huang, Fengjiao Wang, Die Hu, Daoquan Peng, Bilian Yu

**Affiliations:** 1 Department of Cardiovascular Medicine, The Second Xiangya Hospital, Research Institute of Blood Lipid and Atherosclerosis, Central South University, No.139 Middle Renmin Road, Changsha, 410011 Hunan China; 2 Hunan Key Laboratory of Cardiometabolic Medicine, No. 139 Middle Renmin Road, Changsha, 410011 Hunan China; 3 Department of Spine Surgery, The Second Xiangya Hospital, Central South University, NO.139 Middle Renmin Road, Changsha, 410011 Hunan China; 4 FuRong Laboratory, Changsha, 410078 Hunan China

**Keywords:** Experimental models of disease, Dyslipidaemias

## Abstract

Apolipoprotein O (APOO) plays a critical intracellular role in regulating lipid metabolism. Here, we investigated the roles of APOO in metabolism and atherogenesis in mice. Hepatic APOO expression was increased in response to hyperlipidemia but was inhibited after simvastatin treatment. Using a novel APOO global knockout (*Apoo*^*−/−*^) model, it was found that APOO depletion aggravated diet-induced obesity and elevated plasma cholesterol levels. Upon crossing with low-density lipoprotein receptor (LDLR) and apolipoprotein E (APOE) knockout hyperlipidemic mouse models, *Apoo*^*−/−*^ *Apoe*^−/−^ and *Apoo*^*−/−*^ *Ldlr*^−/−^ mice exhibited elevated plasma cholesterol levels, with more severe atherosclerotic lesions than littermate controls. This indicated the effects of APOO on cholesterol metabolism independent of LDLR and APOE. Moreover, APOO deficiency reduced cholesterol excretion through bile and feces while decreasing phospholipid unsaturation by inhibiting NRF2 and CYB5R3. Restoration of CYB5R3 expression in vivo by adeno-associated virus (AAV) injection reversed the reduced degree of phospholipid unsaturation while decreasing blood cholesterol levels. This represents the first in vivo experimental validation of the role of APOO in plasma cholesterol metabolism independent of LDLR and elucidates a previously unrecognized cholesterol metabolism pathway involving NRF2/CYB5R3. APOO may be a metabolic regulator of total-body cholesterol homeostasis and a target for atherosclerosis management.

**Figure:**
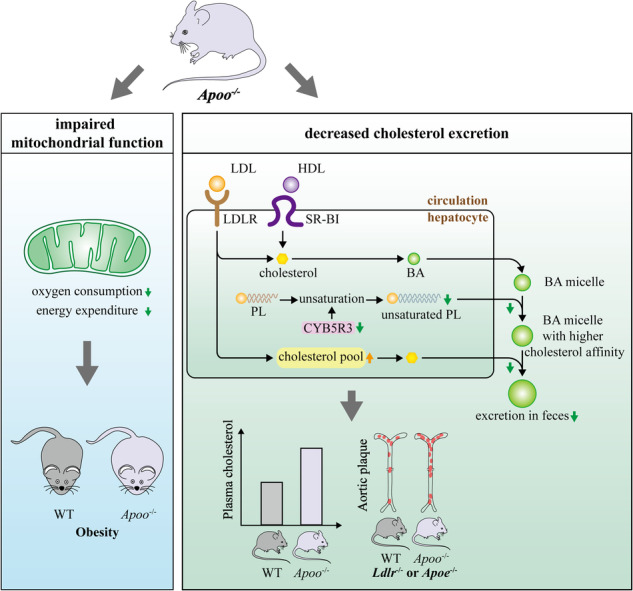
Apolipoprotein O (APOO) regulates plasma cholesterol levels and atherosclerosis through a pathway involving CYB5R3 that regulates biliary and fecal cholesterol excretion, independently of the LDL receptor. In addition, down-regulation of APOO may lead to impaired mitochondrial function, which in turn aggravates diet-induced obesity and fat accumulation.

Apolipoprotein O (APOO) regulates plasma cholesterol levels and atherosclerosis through a pathway involving CYB5R3 that regulates biliary and fecal cholesterol excretion, independently of the LDL receptor. In addition, down-regulation of APOO may lead to impaired mitochondrial function, which in turn aggravates diet-induced obesity and fat accumulation.

## Introduction

The elevation of low-density lipoprotein (LDL) cholesterol (LDL-C) levels is the only causal risk factor for atherosclerotic cardiovascular disease (ASCVD). Additionally, genetic factors account for approximately 40–60% of phenotypic variation in terms of LDL-C levels [1, 2]. However, a recent large-scale study showed that the known genetic variants identified from familial hypercholesterolemia, including mutations in the proprotein convertase subtilisin/kexin type 9 (*PCSK9*), LDL receptor (*LDLR*) and apolipoprotein B (*APOB*), could only explain 2.5% of severe hypercholesterolemia cases [3]. Furthermore, only 10–20% of the total variance in LDL-C is able to be explained by all common and rare loci identified through extensive genome-wide association studies [4, 5], suggesting that there may be some unidentified genes that influence LDL-C levels.

Apolipoprotein O (APOO) is a unique apolipoprotein because it has two forms: a 55 kDa glycosylated form and a 22 kDa non-glycosylated form (6). The secreted 55 kDa glycosylated apolipoprotein is present primarily in high-density lipoproteins (HDL) in the plasma [6]. Although APOO has been recognized as a cholesterol acceptor in vitro as efficient as apolipoprotein A-I (APOA-I), the overexpression of APOO in transgenic mice carrying the human *APOA1* transgene does not significantly affect HDL-mediated cholesterol transport [7], therefore suggesting that APOO may not be a major modulator of HDL functionality.

In addition to its secreted glycosylated form, its non-glycosylated form has been proposed to be a novel constituent of the mitochondrial contact site and cristae organization system (MICOS) complex [8], a conserved multi-subunit complex that occurs in the inner mitochondrial membrane [9]. A previous study [8] found that APOO downregulation led to changes in the mitochondrial morphology with reduced mitochondrial cristae junction (CJ) numbers and oxygen consumption, therefore suggesting that APOO may regulate the MICOS structure and mitochondrial function. Defects in the MICOS subunits are associated with mitochondrial diseases [10–12]. A pathogenic mutation in *APOO* was discovered in a recent whole-exome sequencing analysis of a family affected by X-linked recessive mitochondrial myopathy [13]. In addition to mitochondrial dysfunction, APOO is implicated in fatty acid metabolism in the myocardium and liver [14, 15], while a novel LDL-C-associated locus has been shown to be associated with *APOOP1*, a transcribed pseudogene associated with APOO, through an array-based association analysis in 1,102 Amish subjects [16]. Although its physiological role is not yet completely understood, these prior studies have implied that APOO could play several important intracellular roles in energy and lipid metabolism.

Therefore, in the present study, we explored the effects of the genetic depletion of *Apoo* on total-body energy homeostasis and lipid metabolism, especially hepatic cholesterol metabolism and atherosclerosis in mice. This is the first in vivo experiment to verify the effect of APOO on cholesterol regulation independent of LDLR, and to clarify the previously unrecognized cholesterol metabolism pathway involving NRF2/CYB5R3. Overall, our data provide insights into the roles of APOO as a metabolic regulator of energy and in total-body cholesterol homeostasis.

## Material and methods

Full expanded methods are available in the Supplementary Material.

### Mice and treatments

Leptin-deficient ob/ob male mice, along with their littermate WT counterparts were purchased from Hunan SJA Laboratory Animal Co., Ltd. from a colony derived from the Jackson Laboratory and *Apoo*^*−/−*^ mice were purchased from the Knockout Mouse Project (*Apoo*^*tm1a(KOMP)Wtsi*^, cat. no. 046638-UND; KOMP, University of California, Irvine, CA, USA). The *Apoo* knockout-first, reporter-tagged insertion allele encodes a promoter-driven selection cassette and three loxP and two FRT sites. We then identified mice carrying floxed alleles of *Apoo* (*Apoo*^*flox/flox*^) from *Apoo*^*tm1a(KOMP)Wtsi*^ mice via Flp recombinase-mediated removal of selectable markers flanking the Flp recognition target. Liver-specific *Apoo*-deficient mice (*Alb*^*Cre+/-*^ *Apoo*^*flox/flox*^, abbreviated as *Alb*^*Cre*^*Apoo*^*fl/fl*^) were generated by crossing *Apoo*^*flox/flox*^ mice with *Alb*^*Cre+/-*^ mice (cat. no. 035593; Jackson Laboratory, USA). *Ldlr*^−/−^ (cat. no. T001464; GemPharmatech Co., Ltd., China) and *Apoe*^−/−^ (cat. no. 002052; Jackson Laboratory) mice were bred with *Apoo*^*−/−*^ mice to produce *Apoo*^*−/−*^*/Ldlr*^*−/−*^ and *Apoo*^*−/−*^*/Apoe*^*−/−*^ dKO mice. All mice were generated on pure C57BL/6 backgrounds, and these transgenic mice were confirmed by genotyping genomic DNA and a polymerase chain reaction-based method. Primer sequences used in genotyping are given in Table S1.

All experiments were conducted using littermates. All mice were kept in temperature-controlled cages at 22 ± 1 °C with a 12:12 h light/dark cycle. The 8-week-old mice were randomly divided into normal chow diet (NCD, cat. no. MD17121; Medicience Ltd, China), high-fat diet (HFD; cat. no. D12492; Research Diets Inc., USA) with 60 kcal% fat, or cholesterol-containing western diet (HCD; cat. no. D12079B; Research Diets Inc., USA) with 40 kcal% fat and 0.15% cholesterol. Metabolic rates were measured during the 12:12 h light-dark cycle at 23 °C using a comprehensive laboratory animal monitoring system (Columbus Instruments, USA).

All experiments involving animals were reviewed and approved by the Institutional Animal Care and Use Committees of the Central South University (No.2018sydw088). All animal procedures conform to the NIH Guide for the Care and Use of Laboratory Animals.

### Atherosclerosis studies

For analysis of atherosclerosis, 8-week-old dKO mice and single-knockout (KO; *Ldlr* or *Apoe* alone) mice were fed an HCD for 20 weeks (*Apoo/Apoe* dKO) or 12 weeks (*Apoo/Ldlr* dKO). At the end of experiments, general anesthesia was induced with an intraperitoneal injection of ketamine hydrochloride (100 mg/kg) and xylazine (5 mg/kg). After loss of consciousness, blood was collected and animals were euthanized via exsanguination by cardiac puncture. Then, all animals were subjected to whole-body perfusion. The whole aorta was micro-dissected for en face plaque area evaluation, whereas aortic roots were embedded in paraffin and used for further analysis.

Entire aortas were stained with ORO for atherosclerosis lesion quantification as previously described and then imaged with a ZEISS Stemi 508 stereomicroscope (Carl Zeiss Microscopy GmbH, Suzhou, Germany). The lesion area was quantified using Image Pro Plus 6.0 software (Media Cybernetics, USA). The lesion area on the aortic roots was measured at three locations (80 μm between sections) on H&E-stained sections. The data are presented as the total lesion area and were used to evaluate the content of collagen fibers in atherosclerotic plaques. The collagen content in plaques was evaluated by Masson staining, and the content of macrophages was assessed with F4/80 staining. Immunofluorescence analysis was used to examine NLRP3 (1:100; cat. no.BA3677, Boster), IL-1b (1:800; GB11113, Servicebio) and GSDMD (1:100; cat. no.20770-1-AP, Proteintech) in aortic root sections.

### Quantification and statistical analysis

Statistical significance was evaluated using GraphPad Prism 9 software (GraphPad Software, Boston, USA). Unless otherwise noted, statistical analyses for all experiments were conducted using a two-tailed unpaired Student’s *t-*test or a one- or two-way analysis of variance (ANOVA) with multiple comparisons. Data are expressed as mean ± standard error of the mean (SEM). Results with a *p* value < 0.05 were considered significant.

## Results

### Expression and characterization of APOO in wild-type (WT; *Apoo*^*+/+*^) and knockout (KO; *Apoo*^*−/−*^) mice

**Fig. 1:**
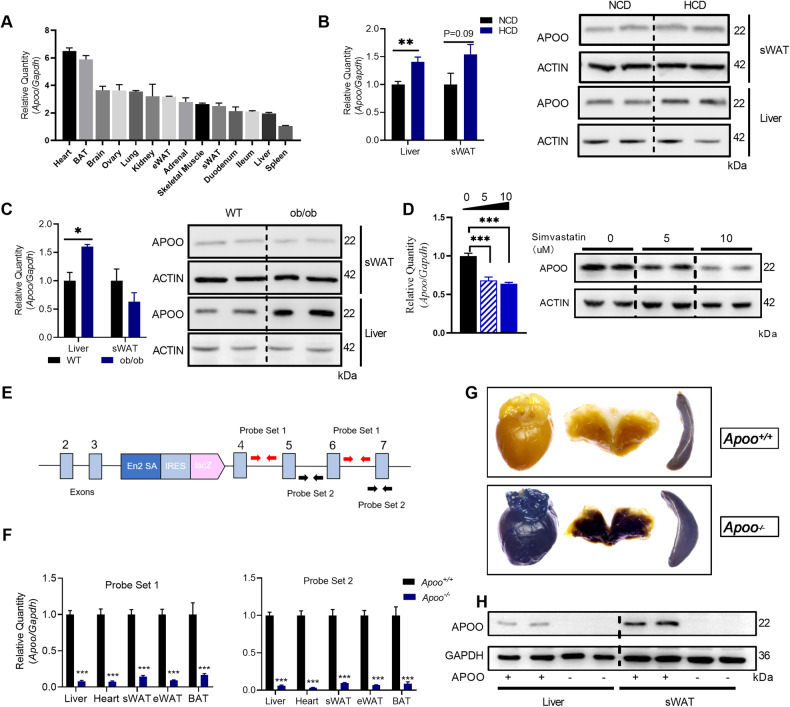
Expression and characterization of APOO in WT and *Apoo*^*−/−*^ mice. **A** Relative mRNA expression of *Apoo* in various tissues from C57BL/6 WT male mice (n = 3). **B** APOO mRNA (left) and protein (right) expression in liver and subcutaneous WAT (sWAT) of C57BL/6 WT male mice, which were fed an NCD or HCD (n = 4) for 12 weeks. **C** APOO mRNA (left) and protein (right) expression in liver and sWAT from 8-week-old male WT control and ob/ob male mice (n = 3) **D** APOO mRNA (left) and protein (right) expression in AML12 cells treated with simvastatin doses of 0, 5, and 10 µM for 24 h (n = 3). **E** Schematic diagram used to create the *Apoo*^*−/−*^ mice, and localization of probe sets used to confirm gene knockout. Red and black arrows indicate the forward and reverse primers used for qPCR verification, respectively. (F-H) 8-week-old male *Apoo*^*+/+*^ and *Apoo*^*−/−*^ mice (n = 6–7) were used for knockout validation. **F** Relative *Apoo* expression in indicated tissues, **G** overnight X-gal staining of selected tissues (heart, WAT, BAT, and spleen), **H** representative immunoblot images of APOO in the liver and sWAT. Values are provided as mean ± SEM. Unpaired two-tailed Student’s t-test (**B**, **C**, **F**) or one-way ANOVA (D). *p < 0.05, **p < 0.01, ***p < 0.001. NCD normal chow diet, ANOVA analysis of variance, SEM standard error of the mean, APOO apolipoprotein O; WT wild-type, HCD high cholesterol diet, WAT white adipose tissue; BAT brown adipose tissue.

*Apoo* mRNA and protein levels were first examined in various tissues collected from C57BL/6 WT males (Fig. 1A and Supplementary Fig. 1A). *Apoo* was detected in several tissues, particularly in mitochondria-rich tissues, such as the heart, brown adipose tissue (BAT), and brain. Compared to the heart and BAT, APOO is less expressed in white adipose tissue (WAT) and liver. To investigate whether APOO expressed in the liver or adipose tissue participated in lipid metabolism, APOO expression was evaluated in the liver and subcutaneous WAT (sWAT) of two dyslipidemia mouse models. Consequently, it was found that APOO expression in the liver was remarkably increased in response to a cholesterol-containing HCD diet and in obese (ob/ob) mice, whereas in the sWAT, APOO expression remained almost unchanged (Fig. 1B, C). In contrast, treatment with simvastatin (a selective inhibitor of cholesterol synthesis) significantly inhibited APOO expression both in AML12 cells (Fig. 1D) and HepG2 cells (Supplementary Fig. 1B), therefore suggesting that hepatic APOO may play essential roles in intracellular cholesterol metabolism.

*Apoo*^−/−^ mice, in which the gene had been ablated using gene trapping with β-galactosidase gene (*lacZ*) insertion into intron 3 of the *Apoo* locus, were used to evaluate the physiological functions of APOO. As shown in Fig. 1E, transcription of the “trapped” *lacZ* reporter gene resulted in a severely truncated APOO protein. Quantitative real-time PCR demonstrated a ~90% reduction in *Apoo* expression within various tissues, including the liver, heart, BAT, and WAT (Fig. 1F). The *LacZ* reporter in the gene-trap cassette of *Apoo*^−/−^ mice (Fig. 1E) enabled X-gal staining as a visual readout of APOO expression in mice carrying a KO allele. Consequently, this approach was used here to confirm the quantitative real-time PCR data. After X-gal staining of indicated tissues from *Apoo*^*+/+*^ and *Apoo*^−/−^ mice, the heart and BAT in *Apoo*^−/−^ mice stained blue while no successful staining was observed in the spleen or WAT (Fig. 1G). This was consistent with the expression patterns of *Apoo* that are illustrated in Fig. 1A. The knockout of APOO in the liver and WAT was also confirmed through protein levels using western blotting (Fig. 1H).

### APOO KO mice exhibited an obesity-prone phenotype with a defect in thermogenesis

**Fig. 2:**
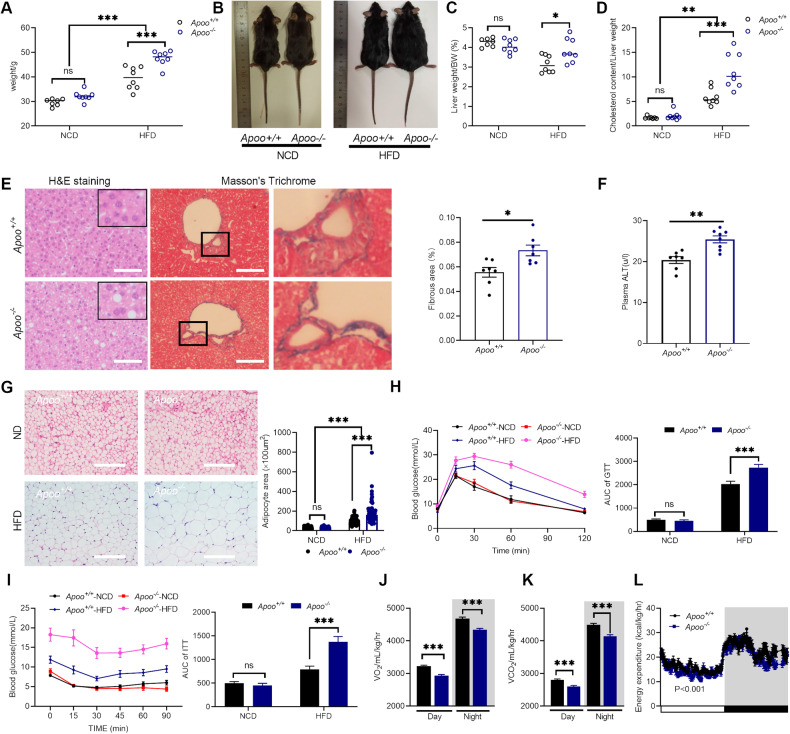
APOO KO mice exhibited an obesity-prone phenotype with a defect in thermogenesis. **A**–**I** Eight-week-old male *Apoo*^*−/−*^ and *Apoo*^*+/+*^ controls were randomly grouped and fed an NCD (n = 7–8) or HFD (n = 8) for 12 weeks. **A** Body weights, **B** Representative images of mice after 12 weeks of NCD or HFD, **C** Liver/body weight ratios, **D** Cholesterol content in the livers, **E** Left: representative hematoxylin and eosin (H&E) and Masson staining of the liver sections of HFD-fed mice, scale bar = 50 μm, boxed regions are shown at a higher magnification; right: quantification of the fibrous area from Masson staining (n = 7), **F** serum ALT levels of HFD-fed mice, **G** Left: representative H&E-stained images of WAT of mice in the indicated groups. Scale bar = 50 μm. Right: quantification of adipocyte area, left: the blood glucose levels during the GTT (**H**) and ITT (**I**) performed on mice in the indicated groups; right: the Area Under the Curve (AUC) of GTT (**H**) or ITT (**I**). Metabolic parameters such as oxygen consumption (VO_2_) (**J**), CO_2_ production (VCO_2_) (**K**), and energy expenditure (**L**) were measured in 10-week-old NCD-fed male mice (n = 8). Values are provided as mean ± SEM. Two-way ANOVA (**A**, **C**, **D**, **G**–**I**, **L**), unpaired two-tailed Student’s t-test (**E**, **F**, **J**, **K**). *p < 0.05, **p < 0.01, ***p < 0.001. NCD normal chow diet, ANOVA analysis of variance, SEM standard error of the mean, APOO apolipoprotein O, HCD high cholesterol diet, WAT white adipose tissue, GTT glucose-tolerance tests, ITT insulin-tolerance tests.

To study the role of APOO in vivo, *Apoo* global KO mice and control littermates were then used to characterize the effects of APOO on metabolism. At the age of 8 weeks, male mice were either switched to a high-fat diet (HFD) or remained on the normal chow diet (NCD), and metabolism parameters were then assessed after 12 weeks. Compared with the corresponding parameters in control littermates, no significant changes in body weight or liver weight to body weight ratio were detected in *Apoo*^−/−^ mice that were on an NCD (Fig. 2A–C). Additionally, the evaluation of fat accumulation in WAT revealed no changes (Fig. 2G). Similar results were also obtained from glucose-tolerance tests (GTTs) and insulin-tolerance tests (ITTs; Fig. 2H, I).

In contrast, *Apoo*^−/−^ male mice showed a similar food intake yet gained more body weight than *Apoo*^*+/+*^ mice when provided with an HFD (Fig. 2A, B, Supplementary Fig. 1C, D). HFD-fed *Apoo*^−/−^ male mice also exhibited higher liver weight-to-body weight ratios in addition to cholesterol accumulation in the liver (Fig. 2C, D). Consistent with fat deposition and fibrosis, as shown in hematoxylin and eosin (H&E) staining and Masson staining (Fig. 2E), alanine aminotransferase (ALT), a marker of hepatic function, was also elevated in HFD-fed *Apoo*^−/−^ male mice (Fig. 2F). In line with hepatosteatosis, fat accumulation in the WAT was also obvious in HFD-fed *Apoo*^−/−^ male mice (Fig. 2G, Supplementary Fig. 1E). Furthermore, glucose clearance and insulin sensitivity were worse in HFD-fed *Apoo*^−/−^ male mice (Fig. 2H, I). We also repeated the phenotype study in female mice, which showed similar trends with HFD-fed *Apoo*^−/−^ male mice (Supplementary Fig. 1F–N).

Furthermore, indirect calorimetry analyses indicated that *Apoo*^*−/−*^ male mice exhibited significantly decreased oxygen consumption (VO_2_), carbon dioxide production (VCO_2_), and energy expenditure (Fig. 2J–L); however, there were no differences in the respiratory exchange ratio (RER), physical activity, or food intake (Supplementary Fig. 1O–Q). This reduced energy expenditure provided a mechanistic explanation for the increased body weight gain and the other metabolic differences observed in these mice when kept on the HFD. In summary, these findings suggested that APOO is required for the regulation of whole-body energy metabolism.

### APOO KO exacerbated lipid profile but had no effect on macrophage-to-feces reverse cholesterol transport (mRCT)

**Fig. 3:**
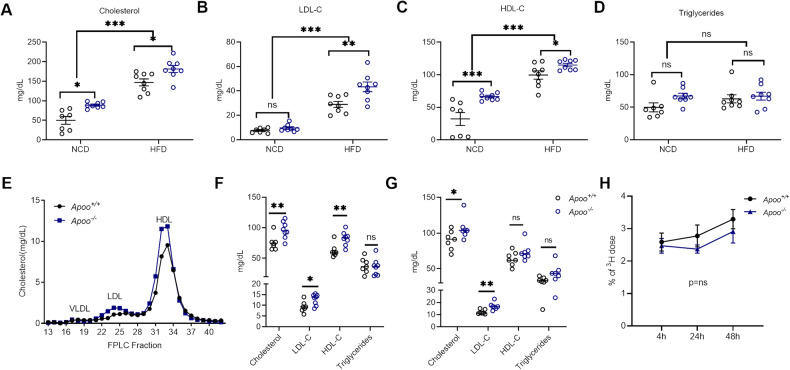
APOO KO exacerbated lipid profile but had no effect on macrophage-to-feces reverse cholesterol transport (mRCT). **A**–**E** Eight-week-old male *Apoo*^*−/−*^ and *Apoo*^*+/+*^ controls were randomly grouped and fed an NCD (n = 7–8) or HFD (n = 8) for 12 weeks, plasma lipid profiles were evaluated. **A** Plasma cholesterol, **B** LDL-C, **C** HDL-C, **D** Triglyceride levels, **E** The pooled plasma from HFD-fed mice was subjected to fast protein liquid chromatography (FPLC) analysis, while cholesterol was measured in each eluted fraction. **F** Eight-week-old female *Apoo*^*−/−*^ and *Apoo*^*+/+*^ controls were fed an HFD for 12 weeks, plasma cholesterol, LDL-C, HDL-C, and triglyceride levels were evaluated (n = 7–8). **G** Eight-week-old male *Apoo*^*−/−*^ and *Apoo*^*+/+*^ controls were fed an HCD for 12 weeks, plasma cholesterol, LDL-C, HDL-C, and triglyceride levels were evaluated (n = 7–8). **H** Time course of ^3^H-cholesterol distribution in the plasma of 8-week-old *Apoo*^*+/+*^ and *Apoo*^*−/−*^ mice provided with NCD. ^3^H-cholesterol–labeled Raw267.4 cells were injected intraperitoneally (5 × 10^5^ cpm/mice). Mice were bled at 4, 24, and 48 h after injection. Values are presented as mean ± SEM. Two-way ANOVA (**A**–**E**, **H**) or unpaired two-tailed Student’s t-test for other panels. *p < 0.05, **p < 0.01, ***p < 0.001. NCD normal chow diet, ANOVA, analysis of variance, SEM standard error of the mean, APOO apolipoprotein O, LDL-C lipoprotein cholesterol, HDL-C high-density lipoproteins cholesterol, HFD high-fat diet.

Blood lipid profiles in all animals were examined next. Interestingly, a 79% and 23% increase in plasma total cholesterol (TC) was observed in male *Apoo*^*−/−*^ mice that were fed an NCD and HFD, respectively (Fig. 3A); which was inconsistent with the fact that other metabolic parameters only exhibited differences in HFD-fed mice. When fed an NCD, all mice showed similar LDL-C levels, while higher HDL-C levels were observed in *Apoo*^*−/−*^ mice (Fig. 3B, C). Additionally, LDL-C and HDL-C levels were markedly higher in *Apoo*^*−/−*^ male mice than in *Apoo*^*+/+*^ mice fed an HFD (Fig. 3B, C). Triglyceride (TG) levels between the two groups were comparable, regardless of diet (Fig. 3D). A fast protein liquid chromatography (FPLC) analysis of pooled sera from HFD-fed mice subsequently confirmed that *Apoo*^*−/−*^ mice had increased circulating levels of cholesterol from LDL and HDL fractions (Fig. 3E). Consistent with male mice, HFD-fed female *Apoo*^*−/−*^ mice also exhibited much higher plasma cholesterol levels, whereas TG levels were comparable with those of *Apoo*^*+/+*^ control littermates (Fig. 3F). To eliminate the effect of obesity on cholesterol levels, plasma cholesterol levels were also evaluated in mice challenged with cholesterol-containing HCD, which contains less fat than HFD. Although body weights of the two groups were comparable after being fed the HCD (Supplementary Fig. 2A), *Apoo*^*−/−*^ mice still exhibited much higher plasma cholesterol levels than *Apoo*^*+/+*^ control littermates (Fig. 3G). In obesity, excessive cholesterol is synthesized and secreted by the hepatocytes [17]. To exclude the possibility that high cholesterol levels after APOO deficiency is secondary to obesity-related excess cholesterol synthesis, we examined the HMG-CoA reductase (HMGCR) expression and cholesterol biosynthesis rate in the liver using ^3^H-labeled H_2_O incorporation assays. The comparable HMG-CoA reductase expression and cholesterol biosynthesis rate in the liver between *Apoo*^*+/+*^ and *Apoo*^*−/−*^ mice (Supplementary Fig. 2B–D) further confirmed the different mechanisms for obesity and hypercholesterolemia. Furthermore, plasma proprotein convertase subtilisin/kexin type 9 (PCSK9), which also participates in dyslipidemia in obesity [18], were comparable between *Apoo*^*+/+*^ and *Apoo*^*−/−*^ mice (Supplementary Fig. 2E).

The secreted 55-kDa glycosylated APOO, which can elicit cholesterol efflux from J774 cells in vitro, has been reported to be present primarily in HDL particles within the plasma. Therefore, to determine the impact of APOO deletion on HDL metabolism and the metrics of mRCT, HDL from the plasma of *Apoo*^*+/+*^ or *Apoo*^*-\-*^ mice that had been fed either a 12-week NCD or HFD were used to elicit cholesterol efflux. There were no significant differences observed in HDL-mediated cholesterol efflux capacity between *Apoo*^*+/+*^ and *Apoo*^*-\-*^ mice, regardless of diet (Supplementary Fig. 2F), which was in line with the results of a previous in vivo overexpression study [7]. Furthermore, the overall in vivo mRCT was also determined. It was found that the presence of ^3^H-tracer was not increased in the plasma, liver, bile, or feces (Fig. 3H and Supplementary Fig. 2G–I), suggesting that genetic ablation of APOO had no impact on HDL cholesterol efflux and mRCT. Both ABCA1 expression in primary peritoneal macrophages and plasma APOA-I levels were comparable between *Apoo*^*+/+*^ and *Apoo*^*-\-*^ mice (Supplementary Fig. 2J–K).

Intestinal dietary cholesterol absorption is a determinant of plasma cholesterol levels. Therefore, a short-term acute cholesterol absorption assay was conducted here to rule out the possibility that altered dietary cholesterol uptake may have contributed to increased plasma cholesterol levels in *Apoo*^*−/−*^ mice. After oral administration of radio-labeled cholesterol, the amount of ^3^H-cholesterol in the liver, plasma, and jejunum was found to have remained unchanged in both male and female *Apoo*^*−/−*^ mice (Supplementary Fig. 2L, M).

In addition to cholesterol uptake, cholesterol can also be transported directly from the blood into the intestinal lumen, a process termed trans-intestinal cholesterol excretion (TICE). Under standard laboratory conditions, TICE generally accounts for approximately 30% of the fecal neutral sterol output in mice [19, 20]. The expression of genes related to TICE, including *Ldlr*, ATP-binding cassette transporter G5/G8 (*Abcg5/g8*), Niemann-Pick C1-Like 1 (*Npc1l1*), and farnesoid X receptor (*Fxr*), did not change in the intestines of *Apoo*^*−/−*^ mice (Supplementary Fig. 2N).

### APOO deficiency accelerated atherosclerosis in hyperlipidemic mouse models

**Fig. 4:**
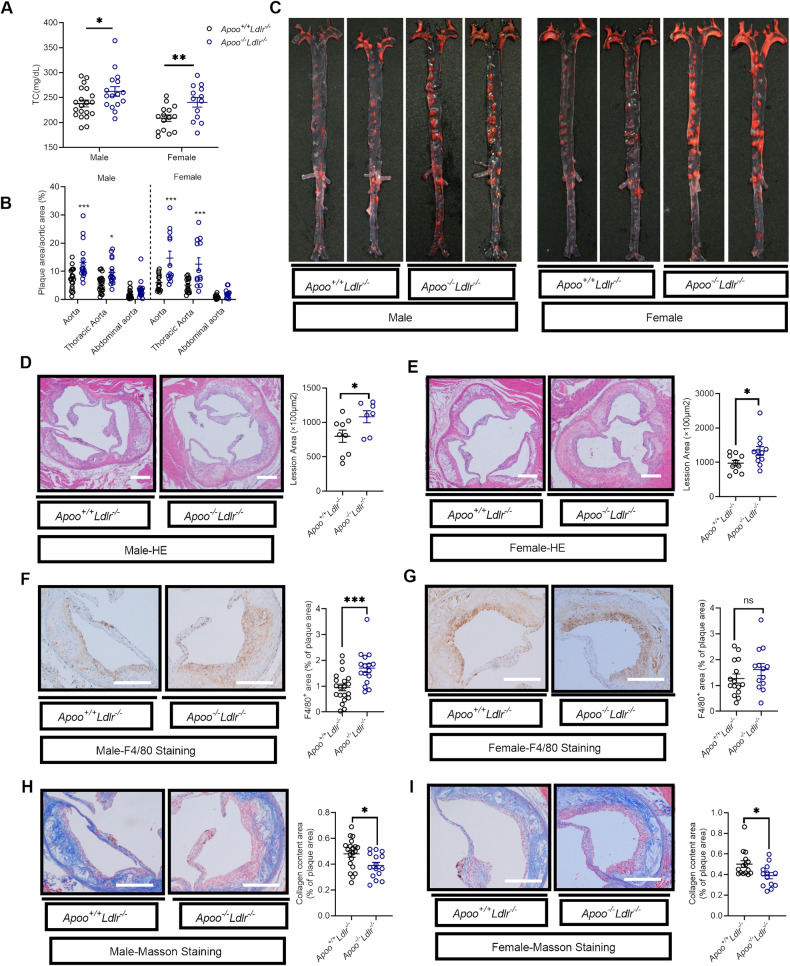
APOO deficiency accelerated atherosclerosis in hyperlipidemic mouse models. Eight-week-old *Ldlr* single knockout (*Apoo*^*+/+*^ *Ldlr*^*−/−*^*)* and *Apoo/Ldlr* dKO (*Apoo*^*−/−*^ *Ldlr*^*−/−*^) mice were randomly grouped (n = 16–20 for male, n = 13–15 for female) and fed an HCD diet for 12 weeks. **A** Plasma TC levels in *Apoo*^*+/+*^*Ldlr*^*−/−*^ and *Apoo*^*−/−*^ *Ldlr*^*−/−*^ male and female mice. **B** Quantification of the aorta, thoracic aorta, and abdominal aorta lesion areas, presented as a percentage area of the entire aorta. **C** Representative oil-red O-stained aortas. (**D**, **E**) H&E staining was used to confirm the atherosclerotic plaque at the aortic root from both male **D** (n = 9 for the male *Apoo*^*+/+*^ *Ldlr*^*−/−*^ group, n = 7 for the male *Apoo*^*−/−*^ *Ldlr*^*−/−*^ group) and female (**E**) (n = 10 for the female *Apoo*^*+/+*^ *Ldlr*^*−/−*^ group, n = 12 for the female *Apoo*^*−/−*^ *Ldlr*^*−/−*^ group) mice. Scale bar = 200 µm. (**F**, **G**) F4/80 staining was used to detect macrophage infiltration at the aortic root plaque in male **F** and female **G** mice. Scale bar = 100 µm. Masson staining was used to determine the collagen composition of the aortic root plaque in male (**H**) and female (**I**) mice. Scale bar = 100 µm. Values are represented as mean ± SEM. Two-way ANOVA (**B**) or unpaired two-tailed Student’s t-test for other panels. *p < 0.05, **p < 0.01, ***p < 0.001. ANOVA analysis of variance, SEM standard error of the mean, APOO apolipoprotein O, HCD high cholesterol diet, H&E hematoxylin and eosin.

LDL cholesterol metabolism through hepatic LDLR-mediated uptake plays a vital role in the maintenance of plasma cholesterol levels. Consequently, it was determined whether the effects of APOO on cholesterol metabolism were dependent on LDLR in *Apoo/Ldlr* double knockout (dKO) mice. At 2 months of age, male and female *Apoo/Ldlr* dKO mice both showed significantly elevated plasma TC levels compared with the corresponding levels in their *Ldlr*^*−/−*^ littermates (Fig. 4A), therefore implying that the effects of APOO on cholesterol metabolism were independent of LDLR.

Next, atherosclerosis was quantified in *Apoo/Ldlr* dKO and control littermates. Mice were fed an atherogenic HCD for 12 weeks, with body weight gain being evaluated. Male and female *Apoo/Ldlr* dKO mice showed only modest body weight gain (Supplementary Fig. 3C, D) alongside significantly accelerated atherosclerosis development (Fig. 4B, C), as established by en face Oil Red O (ORO) staining of the aortic tree. Moreover, the atherosclerosis burden in the thoracic aorta, rather than the abdominal aorta, was significantly increased in *Apoo/Ldlr* dKO mice of both sexes (Fig. 4B). Accordingly, quantification of the cross-sectional plaque area in the aortic sinus showed an increase in lesion burden for *Apoo/Ldlr* dKO mice of both sexes compared with that in the controls (Fig. 4D, E).

We then analyzed the atherosclerotic plaque components. Notably, a remarkably greater F4/80-positive area was observed in male *Apoo/Ldlr* dKO mice, indicating increased macrophage infiltration (Fig. 4F); an increasing trend was observed in female *Apoo/Ldlr* dKO mice (Fig. 4G). Furthermore, Masson-trichrome staining revealed that male and female *Apoo/Ldlr* dKO mice showed significantly lower collagen contents in plaques (Fig. 4H, I), implying plaque instability and vulnerability. However, there were no significant differences between the fluorescence intensity of IL-1b, NLRP3, and GSDMD in plaques (Supplementary Fig. 3A, B).

Body weight and atherosclerosis were also examined on the *Apoe*^*−/−*^ background. Similar to *Apoo/Ldlr* dKO mice, HCD-fed *Apoo/Apoe* dKO female mice experienced a slightly increased weight gain compared with their age-matched single-KO littermates (Supplementary Fig. 3E). Additionally, *Apoo/Apoe* dKO mice were shown to have displayed significantly higher TC levels and increased lesion formation through en face staining (Supplementary Fig. 3F, G), with similar patterns observed in the aortic root compared with control littermates (Supplementary Fig. 3H). However, there were no significant differences in macrophage infiltration and collagen contents at the aortic roots between *Apoo/Apoe* dKO mice and controls (Supplementary Fig. 3I, J).

### APOO deficiency decreased hepatobiliary cholesterol and phospholipid excretion

**Fig. 5:**
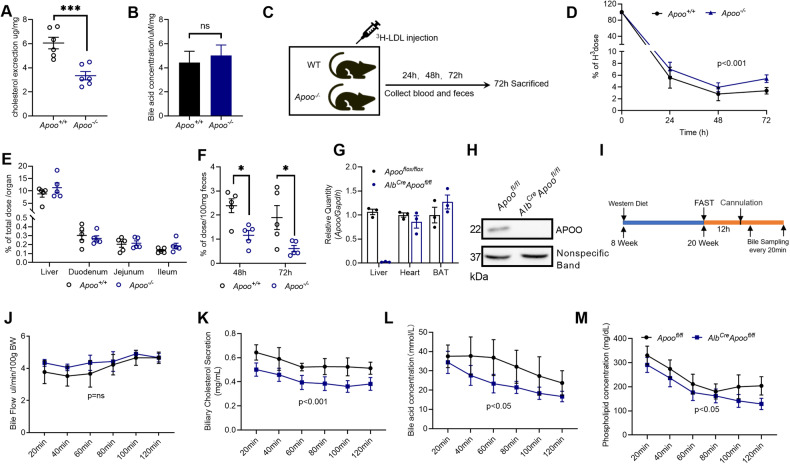
APOO deficiency decreased hepatobiliary cholesterol and phospholipid excretion. Fecal cholesterol (**A**) and bile acid loss (**B**) in 10-week-old male NCD-fed *Apoo*^*−/−*^ mice and their control littermates (n = 6). **C** Schedule of [^3^H]-cholesterol excretion determination in vivo. Radioactivity in plasma (**D**), tissues (**E**), and feces (**F**) after intravenous injection of ^3^H-cholesterol-enriched LDL in 10-week-old male NCD-fed *Apoo*^*−/−*^ mice and their control littermates (n = 5). **G** *Apoo* mRNA expression in the liver, heart, and BAT tissues from *Apoo*^*fl/fl*^ and *Alb*^*Cre*^*Apoo*^*fl/fl*^ mice (n = 3). **H** Western blot of APOO in the livers of *Apoo*^*fl/fl*^ and *Alb*^*Cre*^*Apoo*^*fl/fl*^ mice. (I) Schedule of bile sampling in vivo. Bile flow (**J**), hepatobiliary cholesterol (**K**), hepatobiliary bile acid (**L**), and phospholipid (**M**) output in male *Apoo*^fl/fl^ and *Alb*^Cre^*Apoo*^fl/fl^ mice fed an HFD for 12 weeks (n = 8). Values are represented as mean ± SEM. Two-tailed Student’s t-test (**A**, **B**, **G**) or two-way ANOVA for other panels. *p < 0.05, ***p < 0.001. ANOVA analysis of variance, SEM standard error of the mean, APOO apolipoprotein O, HFD high-fat diet, LDL low-density lipoprotein.

Fecal excretion of endogenous cholesterol (FEEC), the principal pathway of cholesterol elimination, has previously been shown to be negatively correlated with atherosclerosis [21]. To analyze potential changes in fecal cholesterol excretion in *Apoo*^*+/+*^ and *Apoo*^*−/−*^ mice, feces were collected for 24 h. As shown in Fig. 5A, FEEC was significantly decreased in *Apoo*^*−/−*^ mice, whereas bile acid excretion was comparable between *Apoo*^*+/+*^ and *Apoo*^*−/−*^ mice (Fig. 5B). To further confirm the changes in FEEC, native LDL enriched with ^3^H-cholesterol was injected intravenously, followed by the tracer 72 h post-injection (Fig. 5C). Consequently, increased radioactivity was observed in the plasma, indicating delayed substrate clearance from the circulation and/or increased cholesterol secretion (Fig. 5D). Despite the negligible differences in the intestine and liver, radioactivity in feces was significantly decreased in *Apoo*^*−/−*^ mice (Fig. 5E, F).

Once absorbed, cholesterol is partially secreted back into the intestinal lumen via the hepatobiliary pathway. Therefore, we aimed to determine the biliary cholesterol excretion in the liver. To exclude the influence of other tissues on hepatic cholesterol excretion, the bile flow and biliary output of cholesterol, phospholipids, and bile acids in liver-specific *Apoo*-deficient (*Alb*^*Cre*^*Apoo*^*fl/fl*^) mice was then measured. Ablation of APOO in the liver was confirmed at both the mRNA and protein levels (Fig. 5G, H). Despite comparable bile flow, cholesterol output was significantly decreased in *Alb*^*Cre*^ *Apoo*^*fl/fl*^ mice (Fig. 5I–K). Decreased bile acid output (Fig. 5L) suggested the impaired bioconversion of cholesterol into bile acids and/or secretion via the hepatobiliary pathway. However, fecal bile acid excretion remained unchanged (Fig. 5B), suggesting that reduced hepatobiliary bile acid excretion may have been compensated through enterohepatic circulation. In addition to cholesterol and bile acids, the bile of *Alb*^*Cre*^ *Apoo*^*fl/fl*^ mice displayed a lower phospholipid output than that of control *Apoo*^*fl/fl*^ littermates (Fig. 5M), indicating that changes in lipids and phospholipids could potentially explain the decrease in cholesterol excretion.

### APOO regulated the saturation of fatty acyl chains of phosphatidylcholine (PC), which was correlated with cholesterol levels

To explore the mechanism of decreased cholesterol excretion after APOO deficiency, the expression of ABCG5/G8 heterodimer and liver X receptor α (LXRα), that is the key pathway that is responsible for the biliary cholesterol excretion [22], was determined. However, there were no significant differences observed in ABCG5/G8 and LXRα protein expression (Supplementary Fig. 4A), indicating that decreased biliary cholesterol excretion in APOO-deficient mice was not dependent on ABCG5/G8 activity and may instead be caused by other mechanisms.

**Fig. 6:**
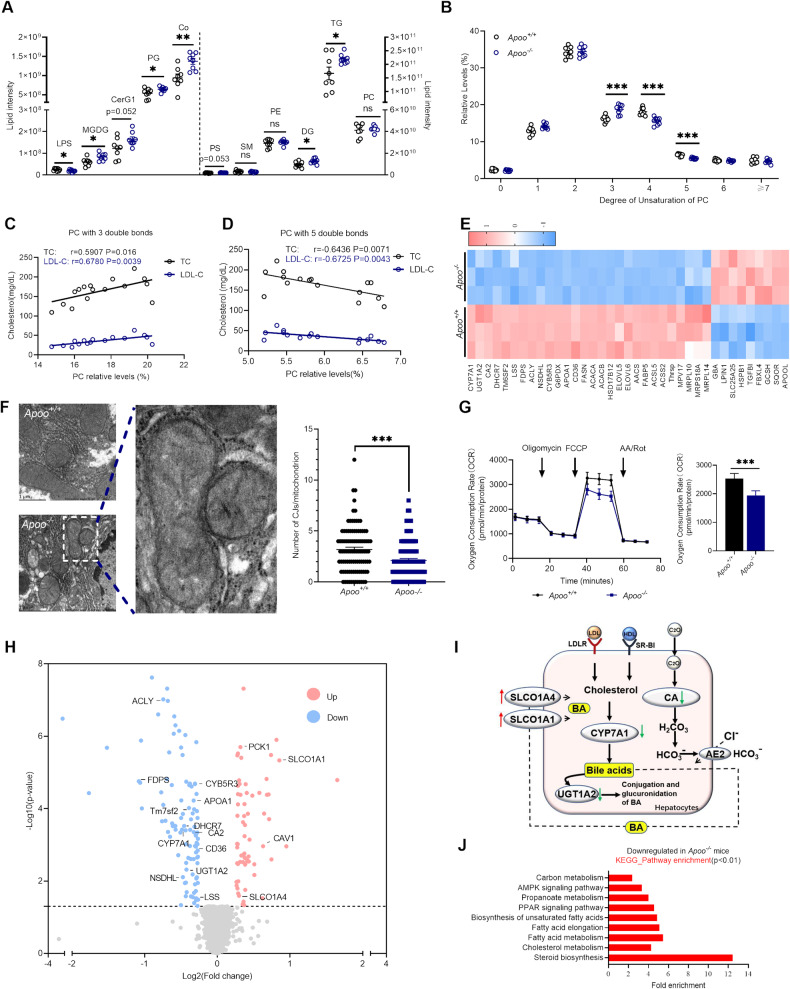
APOO regulated the saturation of fatty acyl chains of phosphatidylcholine (PC), which was correlated with cholesterol levels. **A**–**D** Eight-week-old *Apoo*^*−/−*^ and *Apoo*^*+/+*^ mice were randomly grouped and fed an HFD diet for 12 weeks, liver lipidomics were performed for lipid composition analysis (n = 8). **A** Global abundance of LPS, MGDG, CerG1, PG, Co, PS, SM, PE, DG, TG, and PC revealed by lipidomics, **B**)analysis of fatty acyl composition of PC by the total degree of unsaturation, **C**, **D** correlation levels between the selected lipid species that were analyzed and blood TC or LDL-C levels. **E** The proteome of livers from male HFD-fed *Apoo*^*+/+*^ and *Apoo*^*−/−*^ mice (n = 3) were performed. Heatmap of the abundance profile of proteins related to mitochondrial function was shown. **F** Representative TEM images of the mitochondrion in male NCD-fed *Apoo*^*+/+*^ and *Apoo*^*−/−*^ mice. Quantification represents numbers of CJs in one hundred arbitrarily chosen mitochondria per group. **G** Oxygen consumption rate (OCR) determined by XFe96 Seahorse in hepatocytes from *Apoo*^*−/−*^ and *Apoo*^*+/+*^ mice. Measures of mitochondrial respiration were calculated from the OCR trace. **H** Volcano plot of cholesterol metabolism-related proteins of **E**. **I** Summary of the pathways regulating bile acid metabolism of (**E**); green arrows indicate downregulated proteins, while red arrows indicate upregulated proteins. LDLR low-density lipoprotein receptor, SR-BI scavenger receptor class B type I, CYP7A1 cytochrome P450 family 7 subfamily A member 1, UGT1A2 UDP-glucuronosyltransferase family 1, CA2 carbonic anhydrase 2, SLCO1A1 solute carrier organic anion transporter family member 1A1; SLCO1A4: Solute carrier organic anion transporter family member 1A4; HDL high-density lipoprotein; BA bile acid. **J** Significantly upregulated pathway enriched in differentially expressed proteins of (**E**). Values are represented as mean ± SEM. Unpaired two-tailed Student’s t-test (**A**, **B**), or Mann–Whitney test (**F**) or two-way ANOVA (**G**). *p < 0.05, **p < 0.01, ***p < 0.001. LPS lysophosphatidylserine, MGDG monogalactosyldiacylglycerol, CerG1 glucocerebroside, PG phosphatidylglycerol, Co coenzyme, PS phosphatidylserine; PE phosphatidylethanolamine. DG diglycerides, TG triglyceride, PC phosphatidylcholine, PL phospholipid; TM6SF2 transmembrane 6 superfamily member 2; FDPS farnesyl pyrophosphate synthase; NSDHL sterol-4-alpha-carboxylate 3-dehydrogenase, decarboxylating, LSS lanosterol synthase; DHCR7 7-dehydrocholesterol reductase, cAV1 Caveolin-1, ACLY ATP-citrate synthase.

Polyunsaturated PC and essential phospholipids promote fecal and biliary cholesterol excretion and regression of experimentally induced atherosclerosis [23, 24]. Therefore, a comprehensive lipidome analysis was performed to test phospholipid levels and the degree of saturation of phospholipid fatty acids in the liver. Consequently, over 1509 different lipid species were detected, including 354 TGs, 199 PCs, and 174 phosphatidylethanolamines, among other lipid classes (Supplementary Fig. 4B). Additionally, different clustering patterns between *Apoo*^*−/−*^ and *Apoo*^*+/+*^ mice were shown in Supplementary Fig. 4C. As shown in Fig. 6A, APOO KO significantly increased the levels of coenzyme, monogalactosyldiacylglycerol (MGDG), diglycerides (DGs), and TGs, which was consistent with the increased lipid droplets observed in H&E staining. In addition to phosphatidylglycerol (PG) and lysophosphatidylserine (LPS), no phospholipid classes were significantly different in terms of abundance between *Apoo*^*+/+*^ and *Apoo*^*−/−*^ mice.

Subsequently, a systemic comparison was performed, focusing on the fatty acyl chain profile within PCs, which are important for biliary cholesterol excretion [24]. Our data showed that APOO deficiency resulted in substantial alterations in the fatty acyl chain profiles of PCs, with decreased levels of long-chain fatty acyls containing more than 37 carbons (37C; Supplementary Fig. 4D) and a decrease in poly-unsaturated fatty acyls with 4 or 5 double bonds (Fig. 6B). Additionally, these data suggested that even though phospholipid class totals were comparable, the compositions of PCs differed between the livers of *Apoo*^*+/+*^ and *Apoo*^*−/−*^ mice. The compositions of fatty acid side chains of DGs were also determined here, which is an intermediate in the PC biosynthesis pathway. As shown in Supplementary Fig. 4E, an increase was observed in fatty acyls containing less than 33C, while there was a decrease in long-chain fatty acyls containing more than 39C. Consistent with PCs, a reduction in the degree of unsaturation of fatty acyl chains was also observed in DGs (Supplementary Fig. 4F).

To test whether the unsaturation of fatty acyl chains was correlated with serum TC and LDL-C levels, a correlation analysis was performed between the relative abundance of specific lipid species and levels of TC or LDL-C. It was found that PCs with three double bonds were directly correlated with TC and LDL-C levels (Fig. 6C), whereas PCs with five double bonds were inversely correlated with TC and LDL-C levels (Fig. 6D). Moreover, DGs with two double bonds were directly correlated with TC and LDL-C levels (Supplementary Fig. 4G), while DGs with five or seven double bonds were inversely correlated with TC and LDL-C levels (Supplementary Fig. 4H, I). Overall, these data suggested that the disturbance of fatty acid desaturation may have contributed to defective cholesterol clearance and the hypercholesterolemia phenotype observed in *Apoo*^*−/−*^ mice.

### Loss of APOO altered subsets of proteins involving fatty acid elongation and unsaturation

To gain a global perspective of protein changes associated with altered cholesterol excretion and disturbance of fatty acid desaturation in *Apoo*^*−/−*^ mice, livers from mutant and littermate *Apoo*^*+/+*^ mice challenged with an HFD for 12 weeks were profiled using isobaric tags for relative and absolute quantitation (iTRAQ)-based proteomics (cut-off for differential expression: 1.2-fold change in the iTRAQ ratio). The overall number of differentially expressed proteins in mutant livers was 163, of which 64 were upregulated, while 99 were downregulated. Given the previously established role of APOO in MICOS structure and mitochondrial respiration [8], at first, the present study focused on changes in mitochondrion-related proteins. Heatmaps of the proteins showed significant changes in mutant mice involving mitochondrial function (Fig. 6E), including inner membrane mitochondrial protein MPV17, and mitochondrial ribosomal proteins L10 (MRPL10) and L14 (MRPL14). As previously reported in the literature (8), there was a reciprocal influence of APOO on APOOL levels, which was also confirmed in our data. Despite the compensatory increase of APOOL, the loss of APOO still resulted in significant reductions in CJ abundance (Fig. 6F) and maximal mitochondrial respiration rates in primary hepatocytes of *Apoo*^*−/−*^ mice (Fig. 6G).

The present study then focused on proteomic changes involving sterol and fatty acid metabolism to address the possible molecular events leading to alterations in the cholesterol phenotype. Consistent with the reduced biliary bile acid output, the expression of cytochrome P450 family 7 subfamily A member 1 (CYP7A1) and UDP-glucuronosyltransferase family 1 (UGT1A2), which are related to cholesterol and bile acid bioconversion, and carbonic anhydrase 2 (CA2), an enzyme that catalyzes HCO_3_^-^ production to promote bile acid secretion, were all downregulated in *Apoo*^*−/−*^ mice livers. Additionally, two isoforms of the organic anion transporting protein (OATP) family, namely OATP1A1 (SLCO1A1) and OATP1A4 (SLCO1A4), which are involved in bile acid uptake, were both upregulated (Fig. 6H, I).

Proteomic results did not reveal substantial alterations in proteins related to cholesterol excretion, which was consistent with the results of our western blotting analysis shown in Supplementary Fig. 4A. However, functional and pathway analyses did indicate an association of APOO with unsaturated fatty acid and sterol biosynthesis, as well as fatty acid elongation pathways, which were remarkably downregulated in the livers of APOO-deficient mice compared with *Apoo*^*+/+*^ littermates (Fig. 6J). In addition to well-known targets, such as fatty acid synthase (FASN), ATP citrate lyase, acyl-CoA synthetase long-chain family member 5 (ACSL5), fatty acid-binding protein 5 (FABP5), and acetyl-coenzyme A carboxylase alpha (ACACA), other robust targets emerged here, including lipin 1 and phosphoenolpyruvate carboxykinase 1. Additionally, in accordance with significant alterations in the fatty acyl chain profiles, hydroxysteroid (17-beta) dehydrogenase 12 (HSD17B12), elongation of very long-chain fatty acids protein (ELOVL) 5, ELOVL6, and cytochrome b5 reductase (CYB5R3), which participate in fatty acid elongation and unsaturation, were all downregulated.

In summary, our data showed that loss of APOO led to aberrant mitochondrial architecture and significant changes in the mitochondrion-related proteins, with concomitant repression of OXPHOS and lipid dysmetabolism.

### APOO-regulated cholesterol metabolism was dependent on CYB5R3 in vivo

To further elucidate the molecular mechanism responsible for fatty acid dysmetabolism, several differentially expressed proteins in the proteome were first verified. A targeted, quantitative mass spectrometry approach using parallel reaction monitoring (PRM) was used for subsequent validation [25]. The relative abundance data for 12 proteins are presented in Supplementary Fig. 5A. ACACA, FASN, FABP5, and CYB5R3 were all significantly decreased in *Apoo*^*−/−*^ livers compared with the corresponding levels in *Apoo*^*+/+*^ livers. Among these, CYB5R3 has been reported to be involved in hepatic fatty acyl chain desaturation and cholesterol metabolism [26]; therefore, it was considered whether CYB5R3 may be a link between fatty acid and cholesterol dysmetabolism after APOO deficiency.

**Fig. 7:**
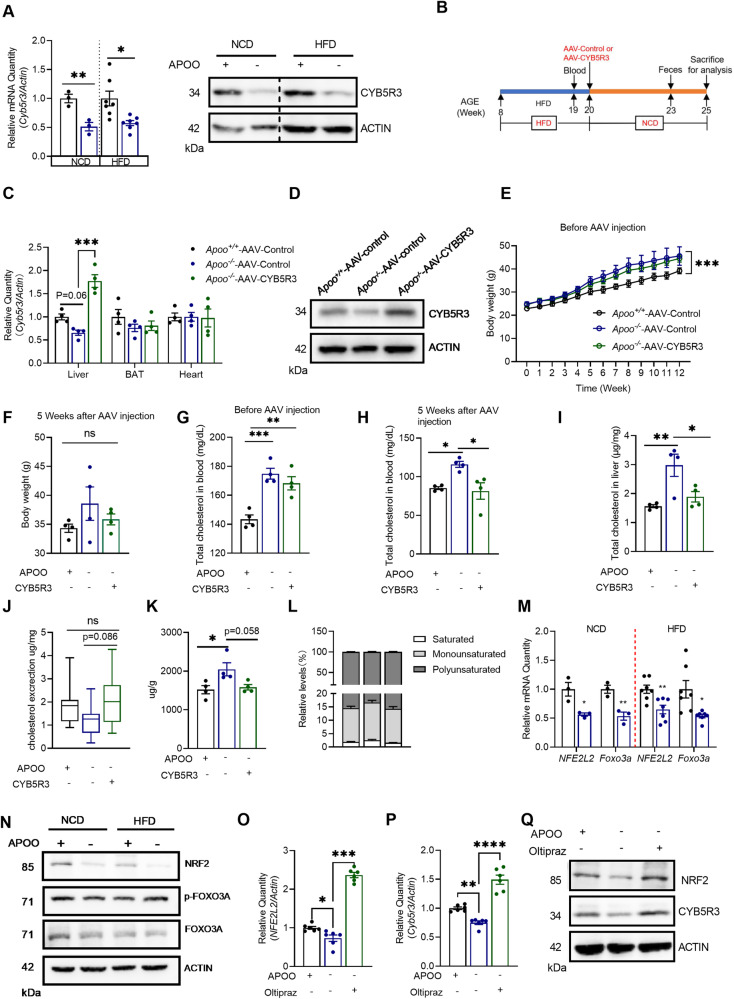
APOO-regulated cholesterol metabolism was dependent on NRF2/CYB5R3 in vivo. **A** CYB5R3 mRNA and protein expression in the livers of mice fed an NCD (n = 3) or HFD for 12 weeks (n = 7). **B**–**L** Eight-week-old *Apoo*^*−/−*^ and *Apoo*^*+/+*^ mice were randomly grouped and both fed an HFD for 12 weeks (n = 4). To restore the expression of CYB5R3 in liver, 50 µl AAV-CYB5R3 or negative control (1 × 10^10^ vector genomes (VG) per mouse) was injected via the tail vein. Then, the HFD diet was changed to an NCD for 5 weeks. Finally, all the animals were subjected to analysis. **B** Experiment Scheme, **C** relative quantification of *Cyb5r3* in the liver, BAT, and heart tissues in different groups, **D** representative western blot of CYB5R3 in the livers from different groups, **E** body weight (weekly) in mice from different groups before AAV injection, **F** body weight in mice 5 weeks after AAV injection, **G** total cholesterol levels in mice before AAV injection, **H** total cholesterol levels in mice 5 weeks after AAV injection, **I** the cholesterol content in the livers of mice 5 weeks after AAV injection, **J** fecal cholesterol loss in mice 5 weeks after AAV injection, **K** the total saturated and monounsaturated PC content in the livers of mice 5 weeks after AAV injection, **L** the percentage of saturated, monounsaturated, and polyunsaturated PC in the livers of mice 5 weeks after AAV injection. **M** Relative quantification of *NFE2L2* and *Foxo3a* in the livers of mice fed an NCD (n = 3) and HFD for 12 weeks (n = 7). **N** Western blot of NRF2, FOXO3A, and p-FOXO3A in the livers of mice fed an NCD (n = 3) and HFD for 12 weeks (n = 7). **O**, **P** Relative quantification of *NFE2L2* and *Cyb5r3* in primary hepatocytes treated with or without 50 µM Oltipraz. **Q** Western blot of NRF2 and CYB5R3 in primary hepatocytes treated with or without 50 µM Oltipraz. Values are mean ± SEM. Unpaired two-tailed Student’s t-test (A,M), or one-way ANOVA for other panels. *p < 0.05, **p < 0.01, ***p < 0.001. ANOVA analysis of variance; SEM standard error of the mean, APOO apolipoprotein O, HCD high cholesterol diet, HFD, high-fat diet; NCD normal chow diet; AAV adeno-associated virus.

First, decreased CYB5R3 gene and protein expression were confirmed in both livers of *Apoo*^*−/−*^ mice with an NCD and HFD (Fig. 7A). Thereafter, CYB5R3 expression was restored in the livers of *Apoo*^*−/−*^ HFD mice following the injection of an adeno-associated virus serotype 2/8 (AAV2/8) expressing CYB5R3 with a thyroxine-binding globulin (TBG) promoter (1 × 10^10^ vector genomes (VG) per mouse) (Fig. 7B). As shown in Fig. 7C, APOO gene expression in *Apoo*^*−/−*^ HFD mice was significantly overexpressed after AAV injection. In contrast, CYB5R3 overexpression was hardly detected in the brown adipose tissue (BAT) or heart. The augmented protein expression of CYB5R3 in the livers of *Apoo*^*−/−*^ HFD mice was also confirmed by western blotting, as shown in Fig. 7D. Although the body weights of *Apoo*^*−/−*^ mice were comparable to *Apoo*^*+/+*^ mice, due to the diet transformation to NCD after AAV injection, CYB5R3 overexpression in *Apoo*^*−/−*^ mice did not result in additional weight loss (Fig. 7E, F). Interestingly, the injection of AAV-CYB5R3 reversed cholesterol dyshomeostasis by APOO knockout, which was shown by the decreased plasma and hepatic cholesterol levels (Fig. 7G–I). Consistently, following the restoration of CYB5R3, *Apoo*^*−/−*^ mice showed a strong trend toward increased fecal cholesterol excretion, being very similar to that in *Apoo*^*+/+*^ mice (Fig. 7J). Although there was a comparable level of PC between *Apoo*^*−/−*^ mice injected with AAV-Control and AAV-CYB5R3, respectively (Supplementary Fig. 5B), reduced amounts of saturated and monounsaturated PC were confirmed after restoration of CYB5R3 in *Apoo*^*−/−*^ mice by comprehensive lipidome analyses (Fig. 7K, L). Similar to alterations of body weights, CYB5R3 restoration in *Apoo*^*−/−*^ mice did not result in the amelioration of serum ALT levels and hepatic fat deposition (Supplementary Fig. 5C–E). Overall, our results confirmed the critical role of liver CYB5R3 in cholesterol dyshomeostasis via APOO knockout, but not in obesity.

To further evaluate the mechanism underlying the decrease in CYB5R3 after APOO knockout, the physical interaction between APOO and CYB5R3 was investigated. Although APOO and CYB5R3 are both located in the mitochondria, CYB5R3 was not identified in the product immunoprecipitated by anti-APOO using immunoprecipitation–mass spectrometry (IP–MS) technology (data not shown). Furthermore, no obvious direct interaction between APOO and CYB5R3 in HepG2 cells was demonstrated via co-IP assay (Supplementary Fig. 5F). The expression of CYB5R3 is reported to be transcriptionally regulated by the cooperation of transcription factor NF-E2–related factor 2 (NRF2, encoded by *NFE2L2*) and fork-head box class O 3a (FOXO3a) [27]. Therefore, the present study sought to examine whether the downregulated CYB5R3 after APOO deficiency was governed by NRF2 and FOXO3a. *NFE2L2* and *Foxo3a* expression levels in the livers of both NCD and HFD-fed *Apoo*^*−/−*^ mice were reduced and significantly correlated with *Cyb5r3* expression (Fig. 7M and Supplementary Fig. 5G). However, only NRF2 protein expression was reduced in the livers of both NCD and HFD-fed *Apoo*^*−/−*^ mice, while neither FOXO3a nor phosphorylated FOXO3a was changed (Fig. 7N). When NRF2 was activated by oltipraz in primary hepatocytes from *Apoo*^*+/+*^ or *Apoo*^*−/−*^ mice, the reduced CYB5R3 expression in *Apoo*^*−/−*^ hepatocytes was restored, thereby indicating that the inhibited CYB5R3 expression was regulated by NRF2 (Fig. 7O–Q).

## Discussion

Our study identified that depleting APOO induced an interesting, yet complex, phenotype. *Apoo*^*−/−*^ mice were presented with diet-induced obesity, increased circulating cholesterol levels independent of LDLR activity, and aggravated atherosclerosis in LDLR- or APOE-deficient mice. Although hypercholesterolemia is a common comorbidity of obesity, hypercholesterolemia in the context of APOO deficiency is more likely to arise from a separate pathway distinct from excessive cholesterol synthesis related to obesity. Here, a pathway is also proposed in which APOO deficiency reduces the degree of unsaturation and elongation of phospholipid fatty acids by inhibiting NRF2-dependent CYB5R3 expression, which in turn reduces cholesterol biliary and fecal excretion. Our results indicated that targeting APOO may present an efficient strategy for the treatment of hypercholesterolemia and atherosclerosis, particularly in homozygous familial hypercholesterolemia, for which an efficacious medical treatment strategy is currently unavailable [28].

Plasma cholesterol levels are tightly regulated by cholesterol synthesis, intestinal cholesterol absorption, hepatic uptake, and cholesterol excretion [29–32]. The present study also showed that increased plasma cholesterol in response to APOO deficiency could partly be attributed to decreased cholesterol elimination through bile. Under normal conditions, biliary excretion of cholesterol at the canalicular membrane, which depends on the formation of mixed micelles, is a vital pathway for the removal of cholesterol from the body. Bile acid micelles form within the bile when water follows osmotically. These micelles then obtain PCs via the canalicular phospholipid transporter ABCB4, forming mixed micelles that have an increased affinity for cholesterol, which is then extracted either directly from canalicular cholesterol transporters (mainly ABCG5/G8) or acquired from cholesterol-rich domains in the canalicular membrane [24, 33, 34]. Therefore, PC secretion and the ABCG5/G8 heterodimer located at the canalicular membrane control biliary cholesterol excretion. Furthermore, it was also found that the presence of polyunsaturated fatty acids would increase PCs unsaturation, while promoting cholesterol elimination through bile and feces [35, 36]. Additionally, the fluidity of the canalicular phospholipid membrane is also important for the efficacy of the biliary secretion process [37]. Although the latter was not investigated in detail herein, this pathway was supported by our findings that, in the context of APOO deficiency, decreased PC unsaturation and secretion, which downregulate cholesterol solubility in mixed micelles and the canalicular membrane, cause decreased cholesterol excretion, resulting in hypercholesterolemia and atherosclerosis.

Why APOO knockout does not affect mRCT is worth discussing. Reports on the effect of PC saturation on mRCT are scarce. One study reported that dietary replacement of saturated fat with monounsaturated (MUFA) fat can promote liver-to-feces RCT, which is consistent with our results [38]. However, mRCT is a multi-step process involving both macrophage-to-plasma and liver-to-feces RCT, and is regulated by many factors, including macrophage cholesterol efflux capacity, HDL-C levels, HDL functionality, and hepatic cholesterol trafficking. Therefore, although our results indicate that APOO knockout can lead to impaired intrahepatic cholesterol trafficking, it is necessary to further investigate whether other processes of mRCT compensate for the absence of changes in mRCT in the future.

In the present study, CYB5R3 was identified as a novel candidate for targeting by APOO. Interestingly, CYB5R3 in the liver has previously been causally linked to the accumulation of high levels of long-chain polyunsaturated fatty acids, improved mitochondrial function, and decreased oxidative damage [26, 39]; however, its role in cholesterol homeostasis remains poorly understood. Notably, several single nucleotide polymorphisms in the *CYB5R3* gene are associated with plasma HDL-C levels [https://t2d.hugeamp.org/region.html?chr=22&end=43045398&phenotype=HDL&start=43013846], indicating a role in the regulation of cholesterol metabolism. The present study also revealed that APOO deficiency inhibited CYB5R3 expression and altered PC unsaturation, whereas the restoration of CYB5R3 in vivo by AAV injection reversed the reduced degree of PC unsaturation while decreasing blood cholesterol levels. Therefore, for the first time, these results established a clear link between APOO and CYB5R3 expression, PC unsaturation, and cholesterol metabolism. However, further studies are still necessary to delineate the specific nature of this complex network.

The transcription factor NRF2 is an important regulator of cellular oxidative stress response and mitochondrial function [40]. Recently, it has been reported that NRF2 was involved in hepatic cholesterol excretion, which is consistent with our data [41]. Due to its cytoprotective role, NRF2 is activated by mitochondrial ROS in the context of mitochondrial dysfunction [42]. As evidenced in the literature and our data, APOO depletion led to aberrant mitochondrial architecture and concomitant repression of OXPHOS. However, contrary to expectations, NRF2 was not activated in our study. Instead, the mRNA and protein expression levels of NRF2 were inhibited upon APOO knockout. At present, there are several studies on the mechanism of NRF2 activation and post-translational control involving ubiquitination and protein degradation dependent on Kelch-like ECH associated protein 1 (Keap1) [43], while only one study deals with the mechanism of NRF2 inhibition. It has previously been reported that miR-144 inhibits NRF2 expression through binding to the 3′-UTR of NRF2 [44]. Therefore, it is worth further exploring the reasons behind aberrant NRF2 activation upon APOO depletion in future studies.

We acknowledge strengths and limitations. We demonstrated the *Apoo*^−/−^ mice in the present study exhibited an intriguing, yet complex, phenotype. Based on our findings, we suggest here that APOO may influence plasma cholesterol levels and atherosclerosis through NRF2/CYB5R3-mediated regulation of biliary and fecal cholesterol excretion. Furthermore, APOO KO aggravated diet-induced obesity and fat accumulation, possibly as a result of impaired mitochondrial function and impaired brown fat thermogenesis [45]. Furthermore, as previously reported, in addition to intracellular non-glycosylated form, APOO has a glycosylated secreted form mainly present in HDL. It was not resolved here that what is the primary functional role of glycosylated APOO. Although APOO deficiency has no effect on cholesterol reverse transport in vivo, we cannot exclude the possibility that APOO regulates cholesterol metabolism through its cellular secretory pathway. In conclusion, these findings provided novel insights into a previously unrecognized effect of APOO in metabolism, while simultaneously extending our knowledge of hypercholesterolemia and atherosclerosis treatment.

### Supplementary information

Supplementary Materials

Supplementary figure 1

Supplementary Figure 2

Supplementary figure 3

Supplementary Figure 4

Supplementary figure 5

Supplementary Material-Original full length western blots

## Data Availability

All data generated or analyzed during this study are included in this published article and its supplementary information files.

## References

[1] Mitchell BD, Kammerer CM, Blangero J, Mahaney MC, Rainwater DL, Dyke B (1996). Genetic and environmental contributions to cardiovascular risk factors in mexican americans. The san antonio family heart study. Circulation.

[2] Pilia G, Chen WM, Scuteri A, Orrú M, Albai G, Dei M (2006). Heritability of cardiovascular and personality traits in 6,148 sardinians. PLoS Genet.

[3] Abul-Husn NS, Manickam K, Jones LK, Wright EA, Hartzel DN, Gonzaga-Jauregui C (2016). Genetic identification of familial hypercholesterolemia within a single U.S. Health care system. Science.

[4] Surakka I, Horikoshi M, M Gi R, Sarin AP, Mahajan A, Lagou V (2015). The impact of low-frequency and rare variants on lipid levels. Nat Genet.

[5] Liu DJ, Peloso GM, Yu H, Butterworth AS, Wang X, Mahajan A (2017). Exome-wide association study of plasma lipids in >300,000 individuals. Nat Genet.

[6] Lamant M, Smih F, Harmancey R, Philip-Couderc P, Pathak A, Roncalli J (2006). Apoo, a novel apolipoprotein, is an original glycoprotein up-regulated by diabetes in human heart. J Biol Chem.

[7] Nijstad N, de Boer JF, Lagor WR, Toelle M, Usher D, Annema W (2011). Overexpression of apolipoprotein o does not impact on plasma hdl levels or functionality in human apolipoprotein a-i transgenic mice. Biochim Biophys Acta.

[8] Koob S, Barrera M, Anand R, Reichert AS (2015). The non-glycosylated isoform of mic26 is a constituent of the mammalian micos complex and promotes formation of crista junctions. Biochim Biophys Acta.

[9] Tirrell PS, Nguyen KN, Luby-Phelps K, Friedman JR (2020). Micos subcomplexes assemble independently on the mitochondrial inner membrane in proximity to er contact sites. J Cell Biol.

[10] Genin EC, Plutino M, Bannwarth S, Villa E, Cisneros-Barroso E, Roy M (2016). Chchd10 mutations promote loss of mitochondrial cristae junctions with impaired mitochondrial genome maintenance and inhibition of apoptosis. EMBO Mol Med.

[11] Gödiker J, Grüneberg M, Duchesne I, Reunert J, Rust S, Westermann C (2018). Qil1-dependent assembly of micos complex-lethal mutation in c19orf70 resulting in liver disease and severe neurological retardation. J Hum Genet.

[12] Zeharia A, Friedman JR, Tobar A, Saada A, Konen O, Fellig Y (2016). Mitochondrial hepato-encephalopathy due to deficiency of qil1/mic13 (c19orf70), a micos complex subunit. Eur J Hum Genet.

[13] Benincá C, Zanette V, Brischigliaro M, Johnson M, Reyes A, Valle D (2021). Mutation in the micos subunit gene apoo (mic26) associated with an x-linked recessive mitochondrial myopathy, lactic acidosis, cognitive impairment and autistic features. J Med Genet.

[14] Turkieh A, Caubère C, Barutaut M, Desmoulin F, Harmancey R, Galinier M (2014). Apolipoprotein o is mitochondrial and promotes lipotoxicity in heart. J Clin Investig.

[15] Schmidinger B, Weijler AM, Schneider WJ, Hermann M (2016). Hepatosteatosis and estrogen increase apolipoprotein o production in the chicken. Biochimie.

[16] Montasser ME, O’Hare EA, Wang X, Howard AD, Mcfarland R, Perry JA (2018). An apoo pseudogene on chromosome 5q is associated with low-density lipoprotein cholesterol levels. Circulation.

[17] Ståhlberg D, Rudling M, Angelin B, Björkhem I, Forsell P, Nilsell K (1997). Hepatic cholesterol metabolism in human obesity. Hepatology.

[18] Vekic J, Zeljkovic A, Stefanovic A, Jelic-Ivanovic Z, Spasojevic-Kalimanovska V (2019). Obesity and dyslipidemia. Metabolism.

[19] van der Velde AE, Vrins CL, van den Oever K, Kunne C, Oude ER, Kuipers F (2007). Direct intestinal cholesterol secretion contributes significantly to total fecal neutral sterol excretion in mice. Gastroenterology.

[20] Jakulj L, van Dijk TH, de Boer JF, Kootte RS, Schonewille M, Paalvast Y (2016). Transintestinal cholesterol transport is active in mice and humans and controls ezetimibe-induced fecal neutral sterol excretion. Cell Metab.

[21] Lin X, Racette SB, Ma L, Wallendorf M, Dávila-Román VG, Ostlund RJ (2017). Endogenous cholesterol excretion is negatively associated with carotid intima-media thickness in humans. Arterioscler Thromb Vasc Biol.

[22] Groen A, Kunne C, Jongsma G, van den Oever K, Mok KS, Petruzzelli M (2008). Abcg5/8 independent biliary cholesterol excretion in atp8b1-deficient mice. Gastroenterology.

[23] Greten H, Raetzer H, Stiehl A, Schettler G (1980). The effect of polyunsaturated phosphatidylcholine on plasma lipids and fecal sterol excretion. Atherosclerosis.

[24] Sehayek E, Wang R, Ono JG, Zinchuk VS, Duncan EM, Shefer S (2003). Localization of the pe methylation pathway and sr-bi to the canalicular membrane: evidence for apical pc biosynthesis that may promote biliary excretion of phospholipid and cholesterol. J Lipid Res.

[25] Chen IH, Xue L, Hsu CC, Paez JS, Pan L, Andaluz H (2017). Phosphoproteins in extracellular vesicles as candidate markers for breast cancer. Proc Natl Acad Sci USA.

[26] Martin-Montalvo A, Sun Y, Diaz-Ruiz A, Ali A, Gutierrez V, Palacios HH (2016). Cytochrome b(5) reductase and the control of lipid metabolism and healthspan. NPJ Aging Mech Dis.

[27] Siendones E, Santacruz-Calvo S, Martín-Montalvo A, Cascajo MV, Ariza J, López-Lluch G (2014). Membrane-bound cyb5r3 is a common effector of nutritional and oxidative stress response through foxo3a and nrf2. Antioxid Redox Signal.

[28] Defesche JC, Gidding SS, Harada-Shiba M, Hegele RA, Santos RD, Wierzbicki AS (2017). Familial hypercholesterolaemia. Nat Rev Dis Primers.

[29] Zhang YY, Fu ZY, Wei J, Qi W, Baituola G, Luo J (2018). A lima1 variant promotes low plasma ldl cholesterol and decreases intestinal cholesterol absorption. Science.

[30] Lu XY, Shi XJ, Hu A, Wang JQ, Ding Y, Jiang W (2020). Feeding induces cholesterol biosynthesis via the mtorc1-usp20-hmgcr axis. Nature.

[31] Wilund KR, Yu L, Xu F, Hobbs HH, Cohen JC (2004). High-level expression of abcg5 and abcg8 attenuates diet-induced hypercholesterolemia and atherosclerosis in ldlr−/− mice. J Lipid Res.

[32] Krishnamurthy K, Glaser S, Alpini GD, Cardounel AJ, Liu Z, Ilangovan G (2016). Heat shock factor-1 knockout enhances cholesterol 7α-hydroxylase (cyp7a1) and multidrug transporter (mdr1) gene expressions to attenuate atherosclerosis. Cardiovasc Res.

[33] de Boer JF, Kuipers F, Groen AK (2018). Cholesterol transport revisited: a new turbo mechanism to drive cholesterol excretion. Trends Endocrinol Metab.

[34] Wiersma H, Gatti A, Nijstad N, Oude ER, Kuipers F, Tietge UJ (2009). Scavenger receptor class b type i mediates biliary cholesterol secretion independent of atp-binding cassette transporter g5/g8 in mice. Hepatology.

[35] Connor WE, Witiak DT, Stone DB, Armstrong ML (1969). Cholesterol balance and fecal neutral steroid and bile acid excretion in normal men fed dietary fats of different fatty acid composition. J Clin Investig.

[36] Nestel PJ, Havenstein N, Whyte HM, Scott TJ, Cook LJ (1973). Lowering of plasma cholesterol and enhanced sterol excretion with the consumption of polyunsaturated ruminant fats. N Engl J Med.

[37] Andersen JP, Vestergaard AL, Mikkelsen SA, Mogensen LS, Chalat M, Molday RS (2016). P4-atpases as phospholipid flippases-structure, function, and enigmas. Front Physiol.

[38] O’Reilly M, Dillon E, Guo W, Finucane O, Mcmorrow A, Murphy A (2016). High-density lipoprotein proteomic composition, and not efflux capacity, reflects differential modulation of reverse cholesterol transport by saturated and monounsaturated fat diets. Circulation.

[39] Rodríguez-López S, López-Bellón S, González-Reyes JA, Burón MI, de Cabo R, Villalba JM (2020). Mitochondrial adaptations in liver and skeletal muscle to pro-longevity nutritional and genetic interventions: the crosstalk between calorie restriction and cyb5r3 overexpression in transgenic mice. Geroscience.

[40] He F, Ru X, Wen T (2020). Nrf2, a transcription factor for stress response and beyond. Int J Mol Sci.

[41] Akl MG, Li L, Baccetto R, Phanse S, Zhang Q, Trites MJ (2023). Complementary gene regulation by nrf1 and nrf2 protects against hepatic cholesterol overload. Cell Rep.

[42] Esteras N, Abramov AY (2022). Nrf2 as a regulator of mitochondrial function: energy metabolism and beyond. Free Radic Biol Med.

[43] Itoh K, Wakabayashi N, Katoh Y, Ishii T, Igarashi K, Engel JD (1999). Keap1 represses nuclear activation of antioxidant responsive elements by nrf2 through binding to the amino-terminal neh2 domain. Genes Dev.

[44] Kukoyi AT, Fan X, Staitieh BS, Hybertson BM, Gao B, Mccord JM (2019). Mir-144 mediates nrf2 inhibition and alveolar epithelial dysfunction in hiv-1 transgenic rats. Am J Physiol Cell Physiol.

[45] Guo X, Hu J, He G, Chen J, Yang Y, Qin D (2023). Loss of apoo (mic26) aggravates obesity-related whitening of brown adipose tissue via pparα-mediated functional interplay between mitochondria and peroxisomes. Metabolism.

