# Supplementary material for: Apolipoprotein O modulates cholesterol metabolism via NRF2/CYB5R3 independent of LDL receptor
Source: Cell Death Dis. 2024 Jun 3;15(6):389. doi: 10.1038/s41419-024-06778-4 (PMC11148037; doi:10.1038/s41419-024-06778-4)
Supplement: Supplementary file 1 — Supplementary Materials [file 41419_2024_6778_MOESM1_ESM.doc]

**Apolipoprotein O modulates cholesterol metabolism via NRF2/CYB5R3 independent of LDL receptor**

**Supplemental Materials**

Expanded Materials & Methods

References(1-13)

Online Supplement Figures 1-5

Online Supplement Table 1-2

**Expanded Materials & Methods**

**X-gal Staining**

X-gal Staining was conducted according to the protocol described in a previous study[1]. Briefly, tissues were fixed in 2% paraformaldehyde (PFA) for 1 h. After six 30-min washes with PBS, tissues were incubated in an X-gal reaction buffer (PBS containing 5 mM Potassium Hexacyanoferrate, 2 mM MgCl_2_, 5 mM Potassium Hexacyanoferrate trihydrate, 0.01% NP40, 0.1% Deoxycholate, and 1.0 mg/ml X-gal in dimethylformamide) at 37 °C overnight. Tissues were then rinsed six times in PBS before images were obtained with a ZEISS Stemi 508 stereomicroscope.

**Cell culture and primary hepatocyte isolation**

The human hepatocellular carcinoma HepG2 cells were cultured in high-glucose dulbecco's modified eagle medium (DMEM) with 10% FBS (cat. no. 10099141C; Gibco, CA，USA) and 1% penicillin/streptomycin (cat. no. 15140122; Gibco) under standard conditions. The isolation of primary mouse hepatocytes was conducted according to the protocol defined in a previous study [2]. Briefly, primary hepatocytes were extracted from male WT and KO mice by collagenase perfusion and purified by Percoll (cat. no. P4937; Sigma, MO, USA) gradient centrifugation. Viable hepatocytes were seeded with MEM-α plating media containing 4% FBS and assayed immediately.

**Preparation of total RNA, synthesis of cDNA, quantitative real-time PCR**

For tissues, total RNA was extracted using TRIzol (cat. no. 15596026; Invitrogen Inc., CA, USA) and GeneJET RNA purification kits (cat. no. K0731; Thermo Fisher Scientific, USA) following the manufacturer's instructions. For cells, total RNA was extracted directly using GeneJET RNA purification kits. Single-stranded cDNA was synthesized from RNA templates using a First Strand cDNA synthesis Kit (cat. no. K1622; Thermo Fisher Scientific). Afterwards, cDNA samples were subjected to qPCR analyses applying a Bio-Rad Real-time System (Bio-Rad) with the primers given in Table S1. All samples were analyzed in duplicate, and the 15 μL reaction contained 7.5 μL PowerUp™ SYBR® Green Master Mix (cat. no. 1725124; Bio-Rad), 300 nM forward and reverse primers, and 70 ng cDNA. For each gene, relative mRNA abundance was calculated using the 2^−ΔΔCT^ method. To determine APOO gene expression in various mouse tissues, RNA was prepared from the indicated tissue from three female C57BL/6 mice. The tissue with the lowest expression level was set to one (spleen in this instance), and all tissue expression values are reported as relative fold-difference.

**Biochemical assay**

Blood lipid, including TC, LDL-C, HDL-C and TG, aminotransferase determination was conducted at the Core Clinical Chemistry Laboratory of the Second Xiangya Hospital. Fast-performance liquid chromatography (FPLC) was used to determine plasma lipoprotein profiles according to standard procedures. The cholesterol contents in each fraction were measured using the WAKO cholesterol assay kit (cat. no. 294-65801; Wako, Japan).

For liver, biliary and feces cholesterol assays, lipids were extracted with chloroform/methanol (2:1), vacuum dried, and re-dissolved with 5% Triton X-100 in isopropanol. Cholesterol was then quantified with the Wako cholesterol assay kit (Wako, Japan). The concentration of bile acids in bile and feces was evaluated using fluorometric total bile acid assay kits (cat. no. MAK309; Sigma, USA) according to the manufacturer’s instructions.

**Assay of biliary cholesterol and bile acids secretion rate**

Before biliary drainage, mice were starved for 4 h and anesthetized with an intraperitoneal injection of ketamine hydrochloride(100mg/kg) and xylazine(5mg/kg). After incision of the abdominal cavity，the common bile duct was ligated. Bile collection started directly after the surgical cannulation of the gallbladder. Bile flow was determined every 20 min for a total of 120 min. Bile acid, the total mass of biliary lipids, including cholesterol and phospholipids, were determined by respective assay kits.

**Intestine cholesterol absorption assay**

A short-term acute cholesterol absorption assay was conducted according to previous literature [3]. Briefly, after 12 h of fasting but with access to water, the mice were gavaged with 2.5 μCi [^3^H] cholesterol (cat. No. 2737431; ARC Inc., USA) with 0.1 mg unlabeled cholesterol in 200 μl corn oil. After two hours, the mice were euthanized with an intraperitoneal injection of ketamine hydrochloride(100mg/kg) and xylazine(5mg/kg), and cardiac exsanguination, and the plasma, jejunum and livers were harvested and investigated for radioactivity using liquid scintillation counting. The absorption rate was calculated according to the following formula: % cholesterol absorption = [^3^H] in tissues (or plasma)/[^3^H] dosing × 100.

**Assay of** ***de novo* hepatic cholesterol biosynthesis**

*De novo* cholesterol biosynthesis was conducted as previously described [4, 5] with minor adjustments. After 16 h of fasting, mice were fed with a low-fat (10 kcal% fat), high-sucrose diet with minimal cholesterol (cat. no. D12450B; Research Diets Inc., USA) to eliminate the interference from feedback regulation. One hour after intraperitoneal injection of 5 mCi [^3^H]-water (cat. No. NET139; ARC Inc., St. Louis, MO, USA) mice were euthanized with an intraperitoneal injection of ketamine hydrochloride(100mg/kg) and xylazine(5mg/kg), and cardiac exsanguination, and 200-300 mg of liver were harvested and rinsed twice in ice-cold PBS. Liver tissues were then saponified overnight at 70 °C in a 2.2 mL of 50% KOH: 95% ETOH (1:10, v: v) mixture. After extraction in 3 mL hexane and dried in air, the sterols extracted were re-dissolved in 300 μL acetone: ETOH (1:1, v: v) mixture. Overnight at room temperature, sterols were then precipitated with 1 mL of digitonin. The radioactivity in the sediments was evaluated using liquid scintillation counting. The cholesterol biosynthesis rate was calculated as the amount of [^3^H]-water incorporated into sterols per hour per microgram tissue.

**Cholesterol excretion**

For the incorporation of radioactive lipids into lipoproteins, 1.2 mL human LDL (Peking Union-Biology Co.Ltd, China) was incubated with 600 μCi [^3^H]-cholesterol (dried down in a glass vial) for 24 h at 37 ℃ with constant mixing by inversion. After centrifugation (15 min, 5000 *g*) and filtration, the uptake of radioactivity was quantified.

Cholesterol excretion was conducted in male HFD-fed mice as previously described [6] with minor adjustment. Briefly, radiolabeled LDL was diluted with PBS and injected intravenously in mice (2*10^6^ cpm/mouse). Animals had free access to food and water for the following 68 h, blood and feces were collected every 24 h after injection. At the end of the experiment, mice were fasted for 4 h and euthanized with an intraperitoneal injection of ketamine hydrochloride(100mg/kg) and xylazine(5mg/kg), and cardiac exsanguination. Blood, duodenums, jejunum, ileum and livers were harvested and determined using liquid scintillation counting.

***In vitro* HDL cholesterol efflux capacity (CEC) assays**

Firstly, we used heparin sodium/manganese chloride precipitation to prepare apoB-depleted mice plasma in our study [7]. A CEC assay was performed as previously described [8, 9]. Briefly, Raw264.7 cells (ATCC, USA) seeded on 48-well plates were loaded with 1 μCi/mL ^3^H-cholesterol and 25 ug/mL acetylated LDL (Peking Union-Biology Co.Ltd, China) together overnight. After washing twice with PBS, cells were stimulated for 24 h with 8-Bromoadenosine 3',5'-cyclic monophosphate (0.3 mmol/L,cat. No. 858463; Sigma) to up-regulate ATP binding cassette transporter A1 (ABCA1) expression. Cells were then rewashed with PBS and incubated with 2.8% (vol/vol) apoB-depleted mice plasma for 6 h to elicit cholesterol efflux. Acyl-CoA: cholesterol acyltransferase (ACAT) inhibitor Sandoz 58–035 (2 μg/mL; cat. No. S9318; Sigma) was added to medium to inhibit cholesterol esterification. Finally, both the medium and cells attached to plates were collected for further radioactivity determination. CEC was calculated as follows: media counts/(media counts + cells counts) ×100%.

***In vivo* macrophage-to-feces reverse cholesterol transport (mRCT)**

*In vivo* mRCT assays were performed as previously described with minor revisions [10, 11]. Briefly, Raw 264.7 macrophages were radiolabeled with 3 µCi/mL [^3^H] cholesterol and 25 µg/mL acetylated LDL for 48 h *in vitro*. After equilibration with DMEM supplemented with 0.2% BSA for 6 h, cells were pelleted by centrifugation(5min, 1000rpm) and suspended in PBS before intraperitoneal injection (5 x10^5^ cpm/mice). Plasma, liver, bile and fecal recovery of labelled cholesterol was analyzed by liquid scintillation at indicated time points. Results were calculated as a percentage of the injected dose recovered in tissues.

**Glucose tolerance and insulin tolerance tests**

We performed glucose tolerance tests (GTT) and insulin tolerance tests (ITT) according to previous literature [4]. Briefly, after 16 h of fasting, mice were subjected to GTTs via intraperitoneal injection of D-glucose (2 g/kg; G7021; Sigma), and glucose levels were measured using OneTouch (Roche, Switzerland), in samples from the tail vein at indicated time points. One week later, ITTs were conducted by injecting insulin (0.75 IU//kg) intraperitoneally after a 6-h fast.

**Histological and transmission electron microscopy (TEM) analysis**

For histology, liver and adipose tissues were immediately fixed overnight in 4% Paraformaldehyde (PFA) and fatty tissue fixative solution (cat. no. G1119, Servicebio, Wuhan, China), respectively. Tissues were then washed with 70% ethanol, embedded in paraffin, and sliced into 5 μm-thick sections for Masson or H&E staining. The adipocyte areas of H&E-stained sections were analyzed using Image Pro Plus 6.

For ORO staining, frozen liver sections were prepared in O.C.T. Compound. 8 μm-thick sections were stained with 0.5% ORO for 30 min. The red lipid droplets were scanned using Pannoramic MIDI II (3DHISTECH Ltd., Budapest, Hungary).

For morphological analysis of mitochondria, liver tissues harvested from the *Apoo^+/+^* or *Apoo^-/-^* mice were fixed in a 2% glutaraldehyde solution and then minced into 1–2 mm^3^ pieces on ice. Samples were re-fixed using osmic acid for a few hours, and images were captured using a Tecnai G2 Spirit TWIN microscope (FEI, Hillsboro, OR, USA). A hundred mitochondria per group were counted to quantify the number of CJs per mitochondrion.

**Liver lipidomics analysis**

Lipidomics analysis was performed on a Q Exactive plus mass spectrometer coupled to a UHPLC Nexera LC-30A (SHIMADZU, Japan) at Shanghai Applied Protein Technology Co.Ltd. Firstly, lipid extraction from liver tissues was performed according to the methyl tert-butyl ether (MTBE) method. LC-MS/MS analysis was then carried out as previously described[12]. Briefly, reverse phase chromatography was selected for LC separation using CSH C18 column (1.7 µm, 2.1 mm× 100 mm, Waters); mass spectra were acquired by Q-Exactive Plus in positive and negative mode, respectively. Lipid identification (secondary identification), peak extraction and alignment, and quantification were assessed with Lipid Search software version 4.1. The abundance of each fatty acid as a percentage of all side chains was calculated according to previous literature [13].

**Tandem Mass Tagging (TMT) proteomics analysis**

Tandem Mass Tagging (TMT) proteomic analysis of liver samples of female mice fed an HFD for 12 weeks was supported by Jingjie PTM BioLabs. Briefly, after routine protein extraction and trypsin digestion, the tryptic peptides were fractionated by high pH reverse-phase HPLC using Thermo Betasil C18 column. For LC-MS/MS analysis, the tryptic peptides were then dissolved in solvent A, containing acetonitrile (2%) and formic acid (0.1%), and separated via EASY-nLC 1000 UPLC system. Finally, the peptides were subjected to NSI source followed by tandem mass spectrometry (MS/MS) in Q ExactiveTM Plus coupled online to the UPLC. The resulting MS/MS data were processed using the Maxquant search engine (v.1.5.2.8). The Kyoto Encyclopedia of Genes and Genomes (KEGG) database was used to annotate the pathways of the identified proteins. The pathway with a corrected p-value of < 0.05 was considered as significant. Some proteins indicated in the manuscript were validated using parallel reaction monitoring (PRM) in Jingjie PTM BioLabs.

**Immunoblotting**

Protein was extracted from the cells or liver tissues with lysis buffer containing a protease inhibitor cocktail (cat. No. 16829900; Roche Applied Science, USA). The samples were then resolved by 7% or 10% SDS-PAGE, transferred to polyvinylidene fluoride (PVDF) membranes (cat. No. IPVH00010; Millipore, USA) and followed by immunoblot analysis using a specific primary antibody. Antibodies used were as follows: APOO (1:1000; cat. no. MA5-15493; Thermofisher), CYB5R3 (1:000; cat. no. 10894-1-AP, Proteintech), ABCG5 (1:1000; cat. no.27722-1-AP, Proteintech), ABCG8 (1:1000; cat. no. DF6673, Affinity), LXRa (1:1000; cat. no. ab176323, Abcam), NRF2 (1:2000; cat. no.16396-1-AP, Proteintech), FOXO3A (1:1000; cat. no.10849-1-AP, Proteintech), p-FOXO3A (1:1000; cat. no.ab154786, Abcam), β-ACTIN (1:20000; cat. no. AC026, ABclonal), GAPDH (1:1000; cat. no. GB11002, Servicebio), APOA1 (1:1000; cat. no. GB112379, Servicebio), ABCA1 (1:1000; cat. no. DF8233, Affinity Biosciences), Albumin (1:5000; cat. no.16475-1-AP, Proteintech). Subsequently, after washes and incubation with the secondary horseradish peroxidase-coupled antibody, the immune complexes were visualized using a chemiluminescence horseradish peroxidase substrate (cat. no. K-12045; Advansta, USA) and quantified using ImageJ 1.49v software.

**Measurement of mitochondrial respiration**

Mitochondrial respiration was determined using the Seahorse XFe96 Analyzer (Agilent, CA, USA). Isolated primary hepatocytes were cultured in a seahorse plate at a cell density of 90%. To perform the assay, the cells were switched to base medium supplemented with pyruvate (1 mm), glucose (10 mm) and glutamine (2 mm) and preincubated at 37 ℃ without CO_2_ for 1 h. The oxygen consumption (OCR) was measured after injecting oligomycin (4 μm), trifluoromethoxy carbonylcyanide phenylhydrazone (FCCP, 2 μm), and antimycin A plus rotenone (AA/Rot, 1 μm) under basal conditions and normalized by protein content.

**Adeno-Associated Virus (AAV) Infection**

The recombinant adeno-associated virus was constructed by Hanbio Biotechnology Co., Ltd. (Shanghai, China). HBAAV2/8-TBG-3flag-P2A-ZsGreen vector carrying mouse CYB5R3 with a thyroxine-binding globulin (TBG) promoter (AAV-CYB5R3) was generated and an empty vector (AAV-Control) was used as a negative control. To restore the expression of CYB5R3 in the livers of *Apoo^-/-^* mice, 50 µl of AAV-CYB5R3 or the negative control (1 × 10^10^ vector genomes (VG) per mouse) was injected via the tail vein.

**Immunoprecipitation**

rProtein A/G Magnetic IP/Co-IP Kit (cat. no. AM001-01; ACE Biotechnology, Nangjing, China) was used for immunoprecipitation (IP). 10 µg of the targeted antibodies were incubated with protein A/G magnetic beads and incubated at room temperature for 2 h. After washing with 500 µL Lysis/Wash buffer twice, HepG2 cell lysates (500 µg) were added and incubated for another 2 h at room temperature. The complexes that are bound to the protein A/G conjugate were washed and resolved in the SDS-PAGE loading buffer and subjected to immunoblotting or LC-MS/MS analysis.

**References**

1. Bauer RC, Tohyama J, Cui J, Cheng L, Yang J, Zhang X, et al. Knockout of adamts7, a novel coronary artery disease locus in humans, reduces atherosclerosis in mice. Circulation 2015;131:1202-13.

2. Nagarajan SR, Paul-Heng M, Krycer JR, Fazakerley DJ, Sharland AF, Hoy AJ. Lipid and glucose metabolism in hepatocyte cell lines and primary mouse hepatocytes: a comprehensive resource for in vitro studies of hepatic metabolism. Am J Physiol Endocrinol Metab 2019;316:E578-89.

3. Zhang YY, Fu ZY, Wei J, Qi W, Baituola G, Luo J, et al. A lima1 variant promotes low plasma ldl cholesterol and decreases intestinal cholesterol absorption. Science 2018;360:1087-92.

4. Lu XY, Shi XJ, Hu A, Wang JQ, Ding Y, Jiang W, et al. Feeding induces cholesterol biosynthesis via the mtorc1-usp20-hmgcr axis. Nature 2020;588:479-84.

5. Li T, Matozel M, Boehme S, Kong B, Nilsson LM, Guo G, et al. Overexpression of cholesterol 7α-hydroxylase promotes hepatic bile acid synthesis and secretion and maintains cholesterol homeostasis. Hepatology 2011;53:996-1006.

6. Vujić N, Korbelius M, Sachdev V, Rainer S, Zimmer A, Huber A, et al. Intestine-specific dgat1 deficiency improves atherosclerosis in apolipoprotein e knockout mice by reducing systemic cholesterol burden. Atherosclerosis 2020;310:26-36.

7. Davidson WS, Heink A, Sexmith H, Melchior JT, Gordon SM, Kuklenyik Z, et al. The effects of apolipoprotein b depletion on hdl subspecies composition and function. J Lipid Res 2016;57:674-86.

8. Khera AV, Cuchel M, de la Llera-Moya M, Rodrigues A, Burke MF, Jafri K, et al. Cholesterol efflux capacity, high-density lipoprotein function, and atherosclerosis. N Engl J Med 2011;364:127-35.

9. Tang X, Mao L, Chen J, Zhang T, Weng S, Guo X, et al. High-sensitivity crp may be a marker of hdl dysfunction and remodeling in patients with acute coronary syndrome. Sci Rep 2021;11:11444.

10. Mistry RH, Verkade HJ, Tietge UJ. Reverse cholesterol transport is increased in germ-free mice-brief report. Arterioscler Thromb Vasc Biol 2017;37:419-22.

11. Kuwano T, Bi X, Cipollari E, Yasuda T, Lagor WR, Szapary HJ, et al. Overexpression and deletion of phospholipid transfer protein reduce hdl mass and cholesterol efflux capacity but not macrophage reverse cholesterol transport. J Lipid Res 2017;58:731-41.

12. Xu Z, You W, Zhou Y, Chen W, Wang Y, Shan T. Cold-induced lipid dynamics and transcriptional programs in white adipose tissue. BMC Biol 2019;17:74.

13. Saito K, Uebanso T, Maekawa K, Ishikawa M, Taguchi R, Nammo T, et al. Characterization of hepatic lipid profiles in a mouse model with nonalcoholic steatohepatitis and subsequent fibrosis. Sci Rep 2015;5:12466.

**Supplemental Figures and Figure Legends**

**Supplemental Figure 1.** **related to Figure 2**

1. Representative images of APOO in various tissues from C57BL/6 WT male mice.
2. APOO mRNA (left) and protein (right) expression in HepG2 cells treated with simvastatin doses of 0, 5, and 10 µM for 24 h (n=3).
3. Food intake of male *Apoo^+/+^* or *Apoo^-/-^* mice fed with an HFD for 12 consecutive days at the end of intervention (n = 8).
4. Body weight (weekly) in male mice fed an HFD for 12 weeks (n = 8).
5. Representative images of BAT, sWAT, gWAT, and perirenal adipose tissue (PAT) dissected from control and male *Apoo^-/-^* mice on an HFD for 12 weeks.

(F–N) Eight-week-old *Apoo^-/-^* female mice and their *Apoo^+/+^* littermates (n = 7–8) were randomly grouped and fed an HFD for 12 weeks. (F) Body weights (weekly), (G) Representative images of female mice after 12 weeks of an HFD, (H) Liver/body weight ratios, (I) The cholesterol content in the livers, (J) Food intake, (K) Left: representative H&E-stained images of WAT from mice in indicated groups; right: quantification of adipocyte areas in (K) (60 arbitrarily chosen adipocytes were analyzed by ImageJ; scale bar = 50 μm), (L) Left: representative hematoxylin and eosin (H&E) and Masson staining of the liver sections of HFD-fed mice, scale bar = 50 μm, boxed regions are shown at a higher magnification; right: quantification of the fibrous area from Masson staining (n = 7), (M) GTT of mice, (N) ITT of mice.

(O–Q) Respiratory exchange ratio (RER) (O), movement (P), and food intake (Q) of NCD-fed male mice, as determined by metabolic cages (n = 8).

Values are represented as mean ± SEM or median with interquartile range (I).Two-way ANOVA (C, D, F, M, N), unpaired two-tailed Student's t-test (K, Q) or Mann–Whitney test (O). ***P < 0.001, was classified as not significant.

HFD, High-fat diet; NCD, Normal chow diet; ANOVA, Analysis of variance; SEM, Standard error of the mean; WAT, White adipose tissue; GTT, Glucose-tolerance tests; ITT, Insulin-tolerance tests.

**Supplemental Figure 2. related to Figure 3**

(A) Body weight (weekly) in male mice fed HCD for 12 weeks (n = 7–8).

(B) Incorporation of tritium-labelled water into sterol in the livers of *Apoo^+/+^* and *Apoo^-/-^* male HFD-fed mice (n = 6).

(C) HMG-CoA reductase mRNA expression in the livers of *Apoo^+/+^* and *Apoo^-/-^* male HFD-fed mice (n = 8).

(D) The proteome of livers HMG-CoA reductase expression in *Apoo^+/+^* and *Apoo^-/-^* male HFD-fed mice (n = 3).

(E) Plasma PCSK9 levels in the *Apoo^+/+^* and *Apoo^-/-^* male HFD-fed mice (n = 8).

1. HDL cholesterol efflux capacity in NCD (n = 5) and HFD (n = 6–7) fed male mice.

(G–I) ^3^H-cholesterol–labeled Raw267.4 cells were injected intraperitoneally (5 x 10^5^ cpm/mice) into 8-week-old *Apoo^+/+^* and *Apoo^-/-^*mice provided with NCD (n = 6). Radioactivity in liver (G), bile (H), and feces (I) after [intraperitoneal injection](javascript:;) were shown.

(J-K) Representative images of primary peritoneal macrophages ABCA1 and plasma APOA-I in the *Apoo^+/+^* and *Apoo^-/-^* male HFD-fed mice.

(L–M) Amount of ^3^H-cholesterol in plasma, liver, and jejunum of female (L, n = 5) and male (M, n = 5) 10-week-old NCD-fed mice after oral gavage with ^3^H-cholesterol for 2 h.

(N) Expression levels of *Apoo*, *Ldlr*, *Abcg5*, *Abcg8*, *Npc1l1*, and *Fxr* in the intestine from HFD-fed *Apoo^+/+^* and *Apoo^-/-^* male mice (n = 8 mice per group).

Values are represented as mean ± SEM. Unpaired two-tailed Student’s t-test (B-H, N) or two-way ANOVA for other panels. **p < 0.01, ***p < 0.001. *Abcg5:* ATP-binding cassette sub-family G member 5; *Abcg8:* ATP-binding cassette sub-family G member 8; *Npc1l1:* NPC1-like intracellular cholesterol transporter 1; *Fxr:* Farnesoid X Receptor*.*

HDL, High-density lipoproteins; HFD, High-fat diet; NCD, Normal chow diet; ANOVA, Analysis of variance; SEM, Standard error of the mean.

**Supplemental Figure 3****. related to Figure 4**

(A and B) Representative immunofluorescence analysis to detect NLRP3 (green particles), IL-1b (pink particles) and GSDMD (red particles) in frozen aortic root sections from *Apoo^+/+^ Ldlr^-/-^*and *Apoo^-/-^ Ldlr^-/-^* mice (right male, left female), nuclei were stained with DAPI (blue particles). Scale bar=100μm.

(C and D) Body weight (weekly) in male (C) and female (D) *Apoo^+/+^ Ldlr^-/-^*and *Apoo^-/-^ Ldlr^-/-^* mice fed an HCD diet for 12 weeks (n = 16–20 for male, n = 13–15 for female).

(E–J) Eight-week-old female *Apoe* single knockout (*Apoo^+/+^Apoe^-/-^*) and *Apoo/Apoe* dKO (*Apoo^-/-^ Apoe^-/-^*) mice were randomly grouped (n = 13–16) and fed an HCD for 20 weeks. (E) Body weight, (F) Plasma TC and LDL-C levels, (G) Representative oil-red O-stained aortas; the right-hand panel shows the quantification of the aorta, thoracic aorta, and abdominal aorta lesion areas presented as the percent area of the entire aorta, (H–J) Histochemical staining of aortic lesions, (H) Hematoxylin and eosin (H&E) staining, scale bar=200 um, (I) F4/ 80 staining, scale bar=100 um, (J) masson staining and fibrous cap, scale bar=100 um, boxed regions in (J) are shown at higher magnification.

Values are represented as mean ± SEM. Statistical significance was determined using unpaired two-tailed Student’s t-test (F-J) or two-way ANOVA for other panels. *p < 0.05, **p < 0.01

TC, Total cholesterol; LDL-C, Low-density lipoprotein cholesterol.

**Supplemental Figure 4.** **related to Figure 6**

(A) Western blot of ABCG8, ABCG5 and LXRa in the livers of mice fed an HFD for 12 weeks (n = 8).

(B-I) Eight-week-old *Apoo^-/-^* and *Apoo^+/+^* mice were randomly grouped and fed an HFD for 12 weeks, lipidomics were performed for lipid composition analysis (n = 8). (B) distribution of lipid classes considered for subsequent analysis in all the samples detected by LC-MS/MS, (C) the score diagram of orthogonal partial least squares discriminant analysis (OPLS-DA) model; t[1] represents principal component 1, to[1] represents principal component 2, and the ellipse represents the 95 % confidence interval, (D and E) analysis of fatty acyl composition of PC (D) or DG (E) by total carbon chain length, (F) analysis of fatty acyl composition of DG by the total degree of unsaturation, (G–I) correlation levels between selected lipid species analyzed and blood TC or LDL-C levels.

Values are represented as mean ± SEM. Statistical significance was determined using unpaired two-tailed Student’s t-test(D-F). *p < 0.05, **p < 0.01

HFD, High-fat diet; SEM, Standard error of the mean; LDL, Low-density lipoprotein; TC, Total cholesterol; LC-MS/MS, Liquid Chromatography with tandem mass spectrometry.

**Supplemental Figure 5. related to Figure 7**

(A) 12 potential targets selected from proteomic data were validated using parallel reaction monitoring (PRM) in male HFD-fed *Apoo^+/+^* and *Apoo^-/-^* mice (n = 5).

(B-E) Eight-week-old *Apoo^-/-^* and *Apoo^+/+^* mice were randomly grouped and both fed an HFD diet for 12 weeks (n = 4). To restore the expression of CYB5R3 in liver, 50 µl AAV-CYB5R3 or negative control (1 × 10^10^ vector genomes (VG) per mouse) was injected via the tail vein. Then, the HFD was changed to NCD for 5 weeks. Finally, all the animals were subjected to analysis. (B) The PC content in the livers of mice 5 weeks after AAV injection, (C) serum ALT levels in mice 5 weeks after AAV injection, (D) the TG content in the livers of mice 5 weeks after AAV injection, (E) Representative images of Oil Red O staining of the liver samples from indicated groups.

(F) Lysates of HepG2 cells were immunoprecipitated (IP) with CYB5R3 antibody or immunoglobulin G (IgG) and immunoblotted with APOO antibody. IgG, negative-control antibody.

(G) Correlation levels between *Cyb5r3* expression and *Foxo3a or* *NFE2L2* expression in the livers of HFD-mice (n = 7).

Values are mean ± SEM. Unpaired two-tailed Student’s t-test(A), or one-way ANOVA for other panels. *p < 0.05, **p < 0.01.

HFD, High-fat diet; NCD, Normal chow diet; AAV, Adeno-associated virus; ANOVA, Analysis of variance; SEM , Standard error of the mean; APOO, Apolipoprotein O.

**Supplemental Tables**

**Table S1 Primer pair sequences used for mice genotyping and quantitative RT-PCR analysis**

| Gene | Species | Forward primer sequence (5′-3′) | Reverse primer sequence (5′-3′) |
| --- | --- | --- | --- |
| *Apoo* Probe Set1 | Mouse | TGTCCAGTGGGGAGTAGACAA | AGTGATCTGGGCAATGGTGA |
| *Apoo* Probe Set2 | Mouse | GGACTCCTTTTTGCTAGAGG | TATGTACCCTCGTAATCCCC |
| *APOO* | Human | CAGCTCCGACACTATTGCGA | GGAGTCCAATAAGGCCAGCA |
| *GAPDH* | Human | CCATGGGTGGAATCATATTGGA | TCAACGGATTTGGTCGTATTGG |
| *Gapdh* | Mouse | AAGGTCATCCCAGAGCTGAA | AGGAGACAACCTGGTCCTCA |
| *Β-Actin* | Mouse | GAGACCTTCAACACCCCAGC | ATGTCACGCACGATTTCCC |
| *Ldlr* | Mouse | CATATGCATCCCCAGTCTTTG | GCAGTGCTCCTCATCTGACTTG |
| *Cyb5r3* | Mouse | TCGCCCGTCTGGTTCATCTA | GCCTCAGAGGGTACTTGATGTC |
| *Abcg5* | Mouse | CTCCGCGGACTTCTACAACA | CTCCGCGGACTTCTACAACA |
| *Abcg8* | Mouse | ACAGCTTGTTCTCCTCGGAA | CCAGCTCACAGGAGTCTTGG |
| *Fxr* | Mouse | ACTCTCAGAGGTATCAGTCCTGC | CAGAGGTTGAGTCTTTCCCAC |
| *Npc1l1* | Mouse | TGTCCCCGCCTATACAATGG | CCTTGGTGATAGACAGGCTACTG |
| *Hmgcr* | Mouse | TGATGGGAGCTTGCTGTGAG | TAGTGCTGGCCACAAGACAG |
| *NFE2L2* | Mouse | GGACATGGAGCAAGTTTGGC | TCCAGCGAGGAGATCGATGA |
| *Foxo3a* | Mouse | TCACACTACGGCAACCAGAC | TGGGCAGCAAAGGACATCAT |
| *Apoo flox* | Mouse | AGGATAGCTGGGCTGGTGTC | CTGCCACAGGAAGCAAAGAG |
| *AlbCre* | Mouse | GAAGCAGAAGCTTAGGAA GATGG | TTGGCCCCTTACCATAACTG |
| *AdipoqCre* | Mouse | GATTTCGACCAGGTTCGTTC | GCTACCCAGCGTTTTCGTTC |
| *Apoo^-/-^* | Mouse | TCGCCTTCTTGACGAGTTCT | CTGCCACAGGAAGCAAAGAG |
| *Apoe^-/-^* | Mouse | GCCTAGCCGAGGGAGAGCCG | GCCGCCCCGACTGCATCT |
| *Ldlr^-/-^* | Mouse | CTCCCAGGATGACTTCCGAT | CGCAGTGCTCCTCATCTGAC |

**Table S2 Non-standard Abbreviations and Acronyms**

| [Abbreviation](javascript:;) | Full name |
| --- | --- |
| APOO | Apolipoprotein O |
| MICOS | Mitochondrial contact site and cristae organization system |
| CJs | Mitochondrial cristae junctions |
| PCR | Polymerase chain reaction |
| GTTs | Glucose tolerance tests |
| ITTs | Insulin tolerance tests |
| ALT | Alanine aminotransferase |
| mRCT | Macrophage-to-feces reverse cholesterol transport |
| FPLC | Fast protein liquid chromatography |
| TICE | Transintestinal cholesterol efflux |
| FEEC | Fecal excretion of endogenous cholesterol |
| OPLS -DA | The orthogonal projections to latent structures discriminant analysis |
| iTRAQ | isobaric tag for relative and absolute quantitation |
| MRPL10 | Mitochondrial ribosomal protein L10 |
| PRM | Parallel reaction monitoring |
| IP–MS | Immunoprecipitation–mass spectrometry |
| PFA | Paraformaldehyde |
| TEM | Transmission electron microscopy |
| TMT | Tandem Mass Tagging |
| PRM | Parallel reaction monitoring |
| PVDF | Polyvinylidene fluoride |
| OCR | Oxygen consumption |
| AAV | Adeno-Associated Virus |
| TBG | Thyroxine-binding globulin |
| PAT | Perirenal adipose tissue |
| RER | Respiratory exchange ratio |
