# Supplementary figures and images for: Apolipoprotein O modulates cholesterol metabolism via NRF2/CYB5R3 independent of LDL receptor
Source: Cell Death Dis. 2024 Jun 3;15(6):389. doi: 10.1038/s41419-024-06778-4 (PMC11148037; doi:10.1038/s41419-024-06778-4)

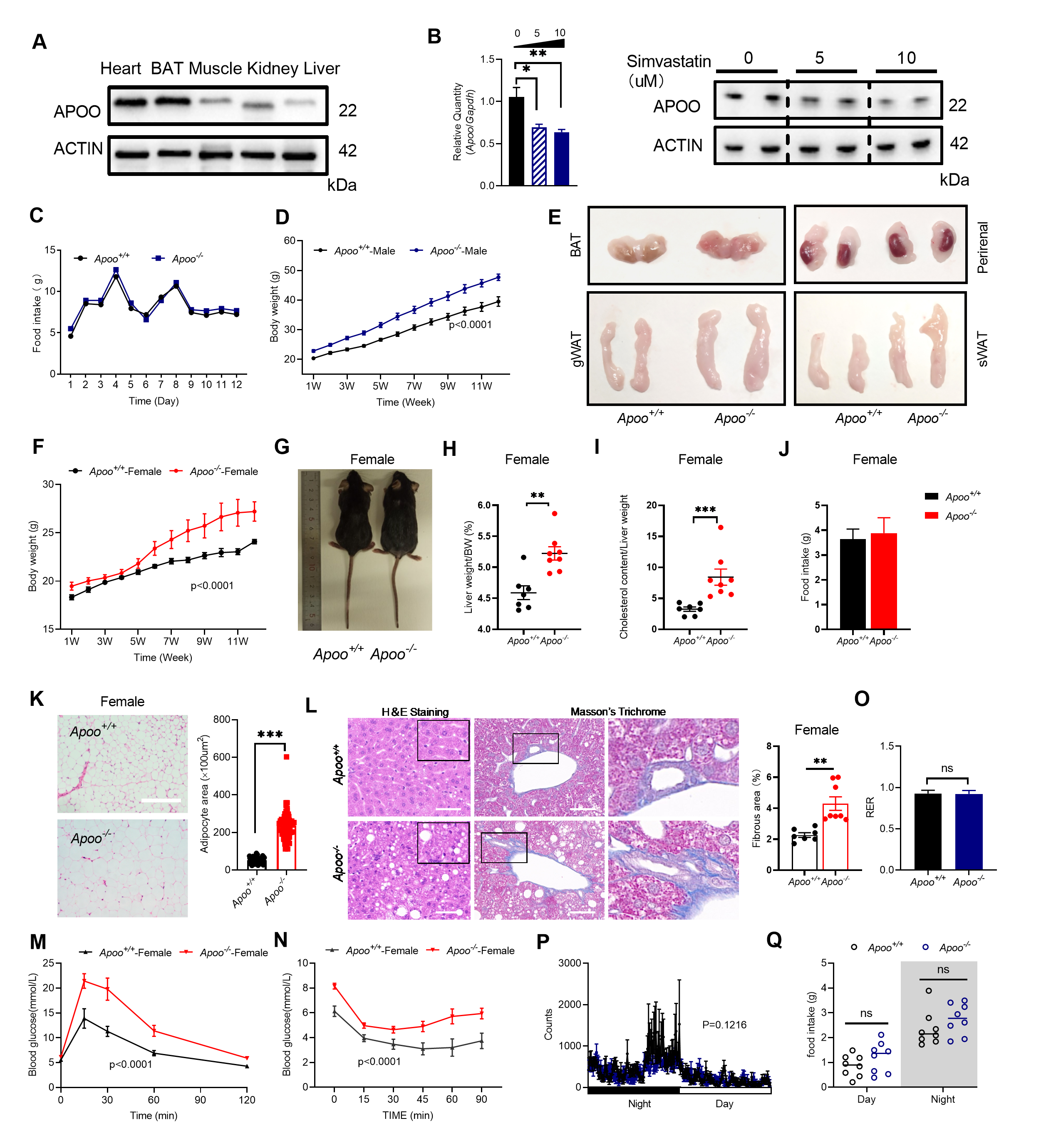

Supplement: Supplementary file 2 — Supplementary figure 1 [file 41419_2024_6778_MOESM2_ESM.tif]

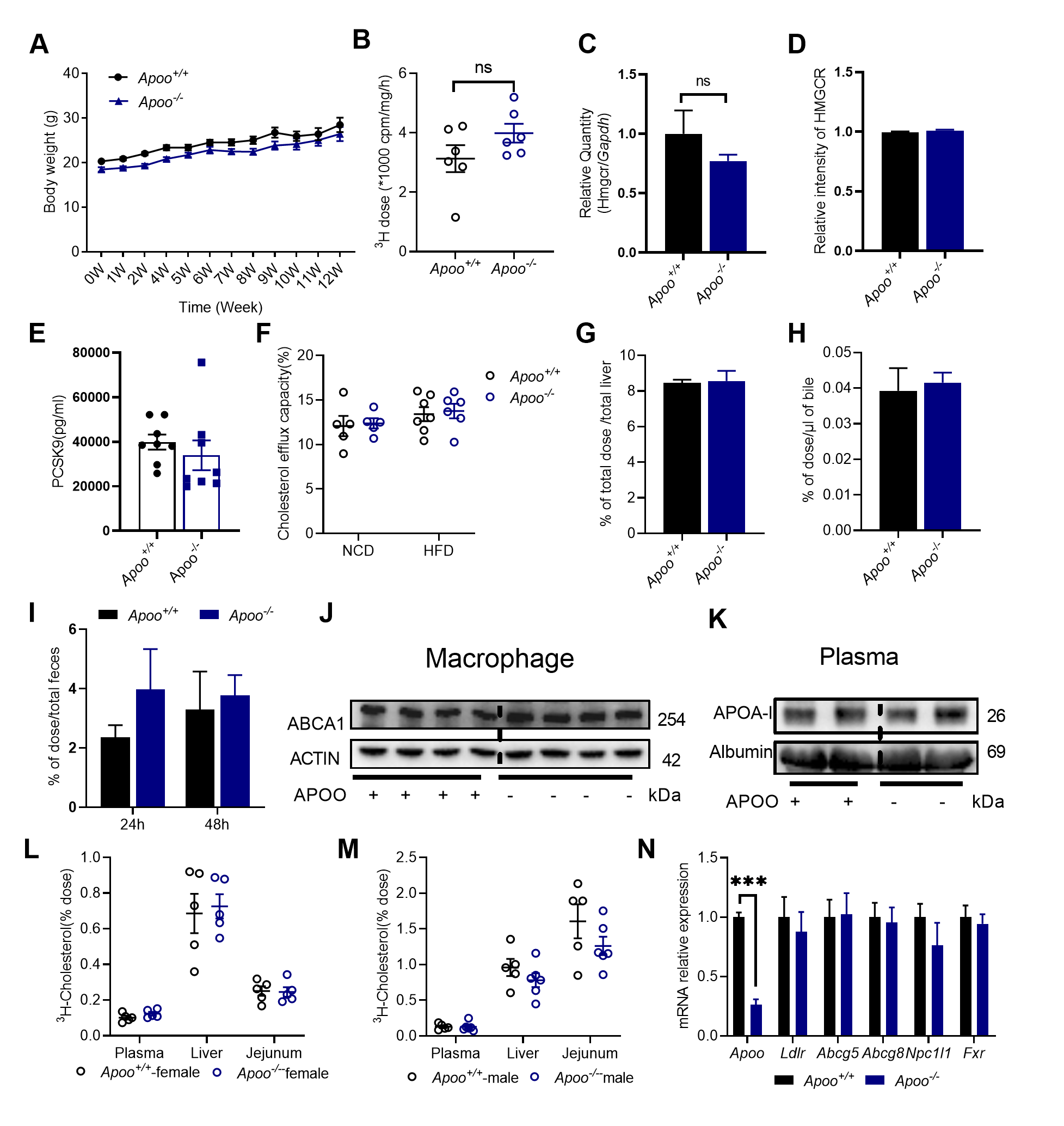

Supplement: Supplementary file 3 — Supplementary Figure 2 [file 41419_2024_6778_MOESM3_ESM.tif]

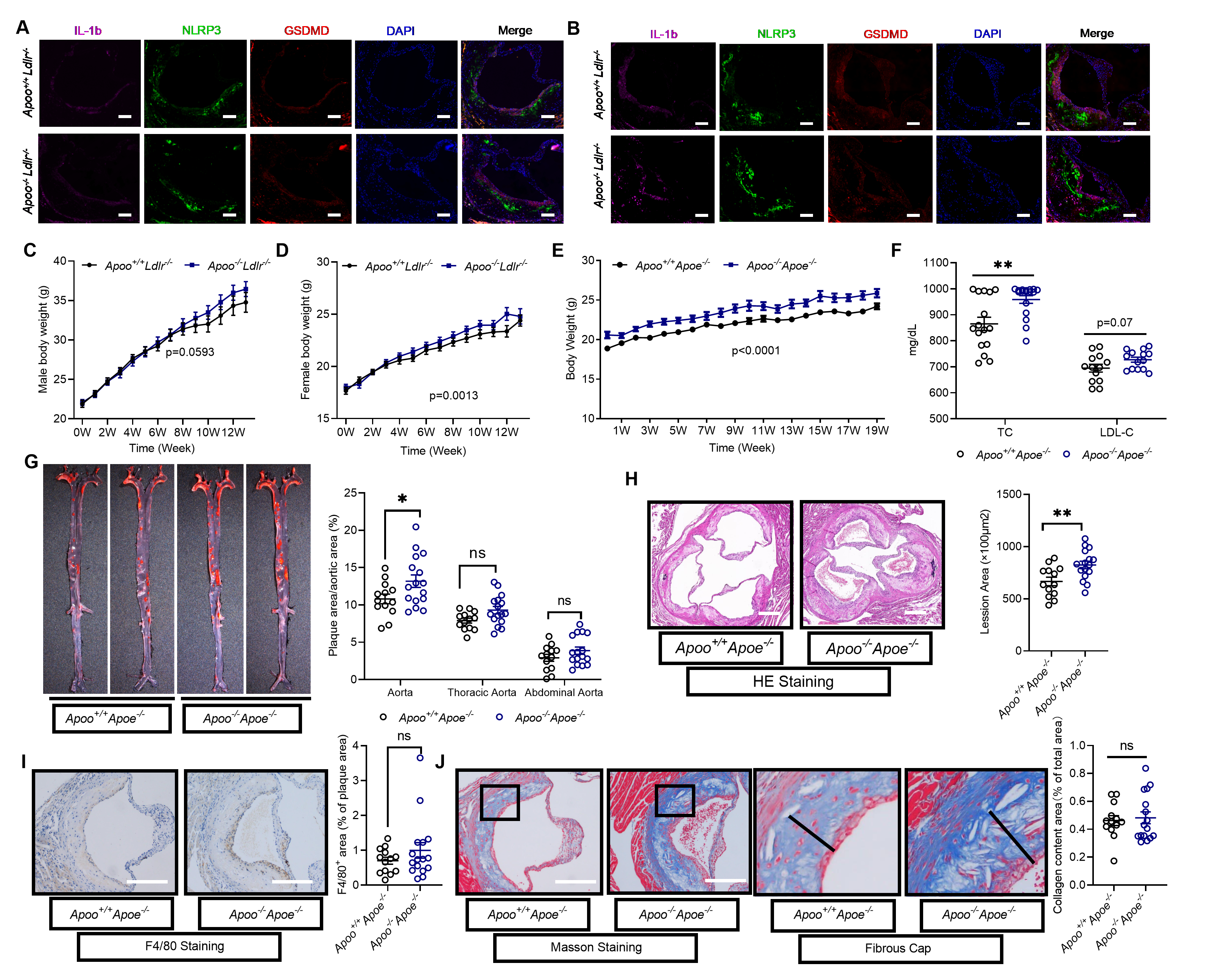

Supplement: Supplementary file 4 — Supplementary figure 3 [file 41419_2024_6778_MOESM4_ESM.tif]

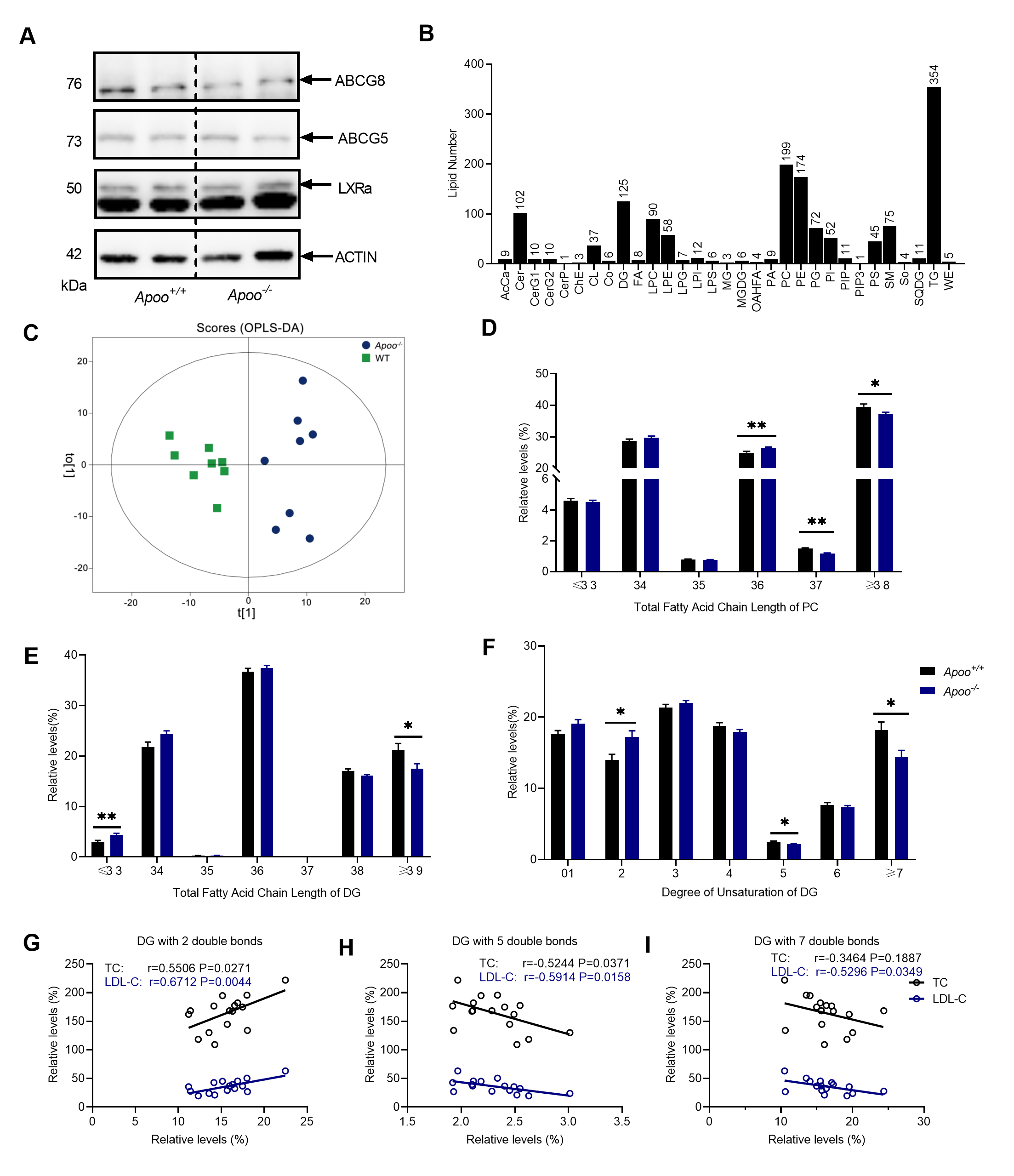

Supplement: Supplementary file 5 — Supplementary Figure 4 [file 41419_2024_6778_MOESM5_ESM.tif]

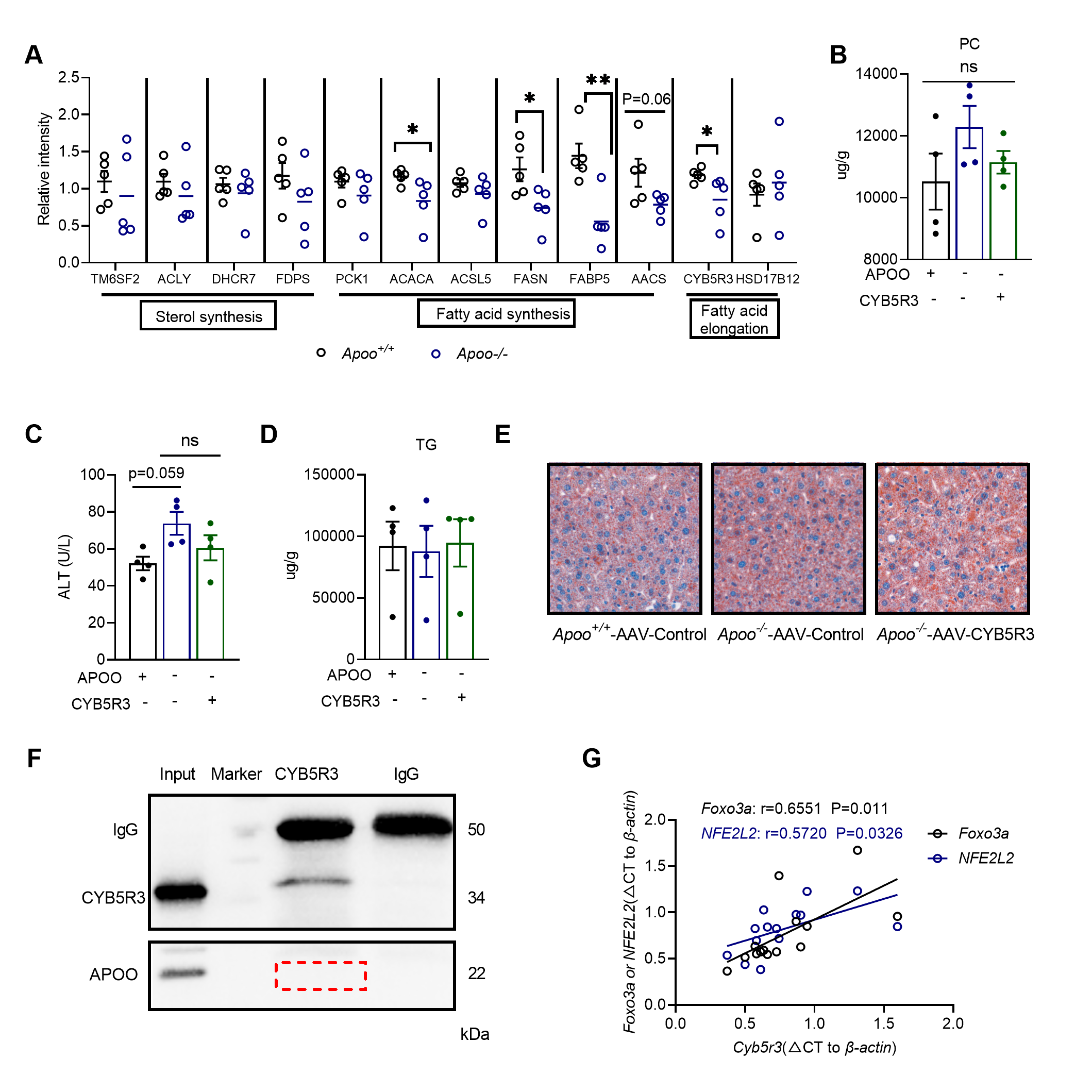

Supplement: Supplementary file 6 — Supplementary figure 5 [file 41419_2024_6778_MOESM6_ESM.tif]
