# Supplementary material for: Apolipoprotein O modulates cholesterol metabolism via NRF2/CYB5R3 independent of LDL receptor
Source: Cell Death Dis. 2024 Jun 3;15(6):389. doi: 10.1038/s41419-024-06778-4 (PMC11148037; doi:10.1038/s41419-024-06778-4)

Figure 1B

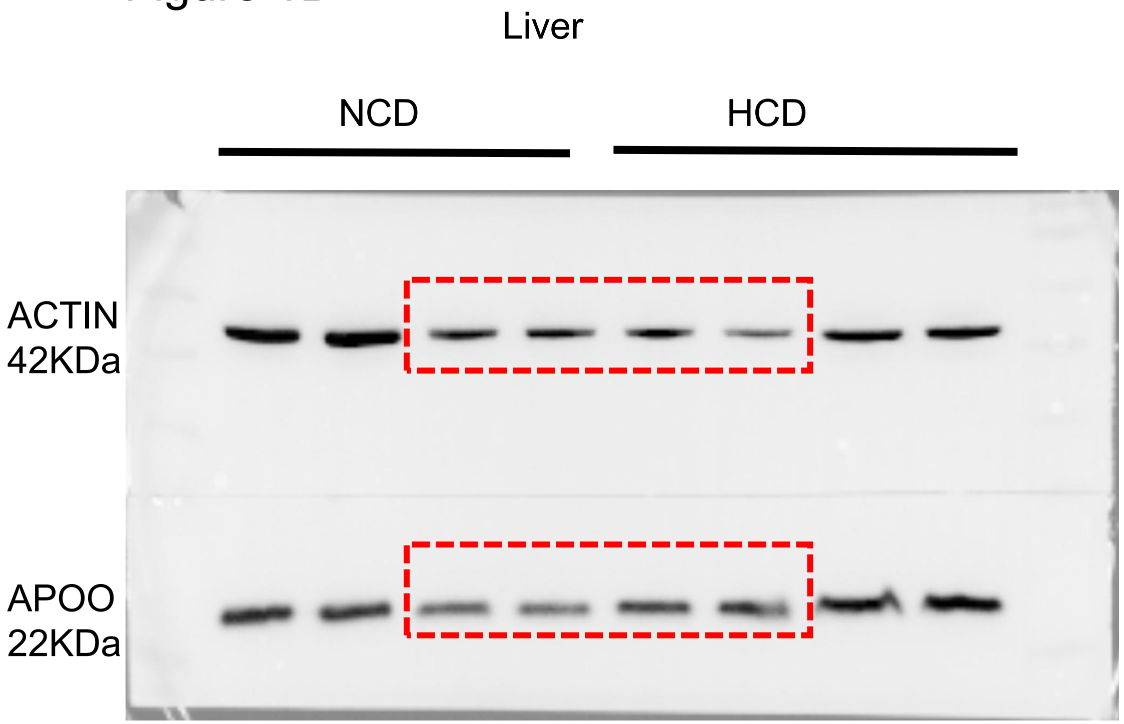

Liver
NCD
HCD
ACTIN
42KDa
APOO
22KDa

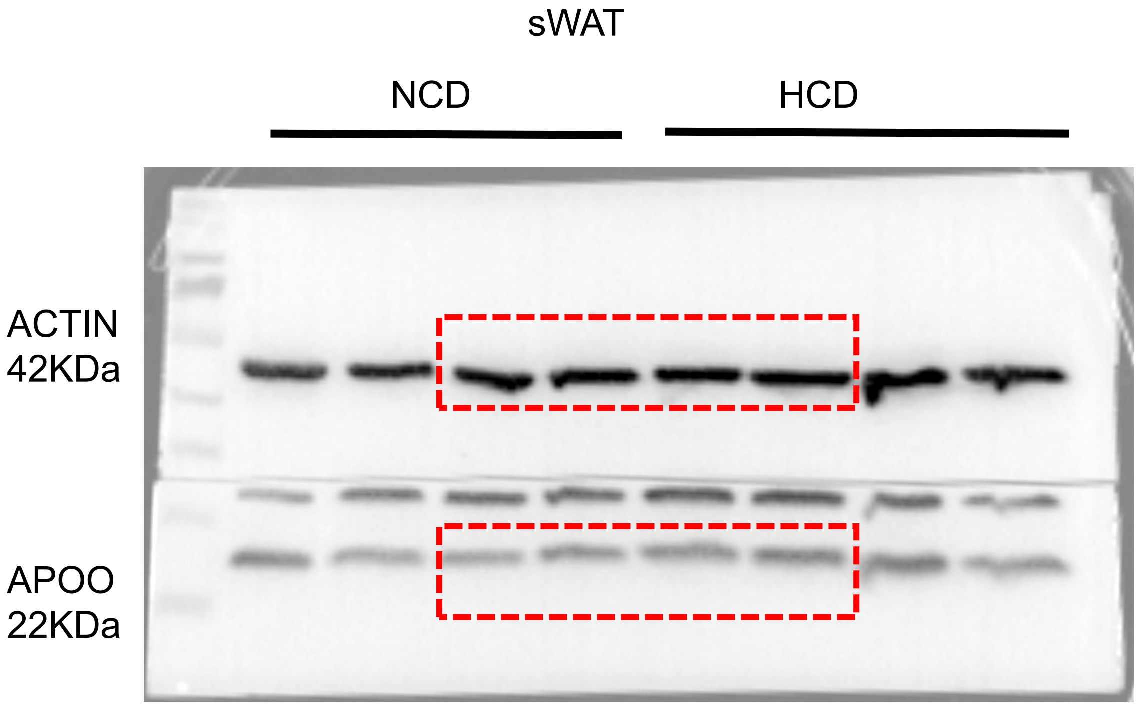

sWAT
NCD
HCD
ACTIN
42KDa
APOO
22KDa

Figure 1C

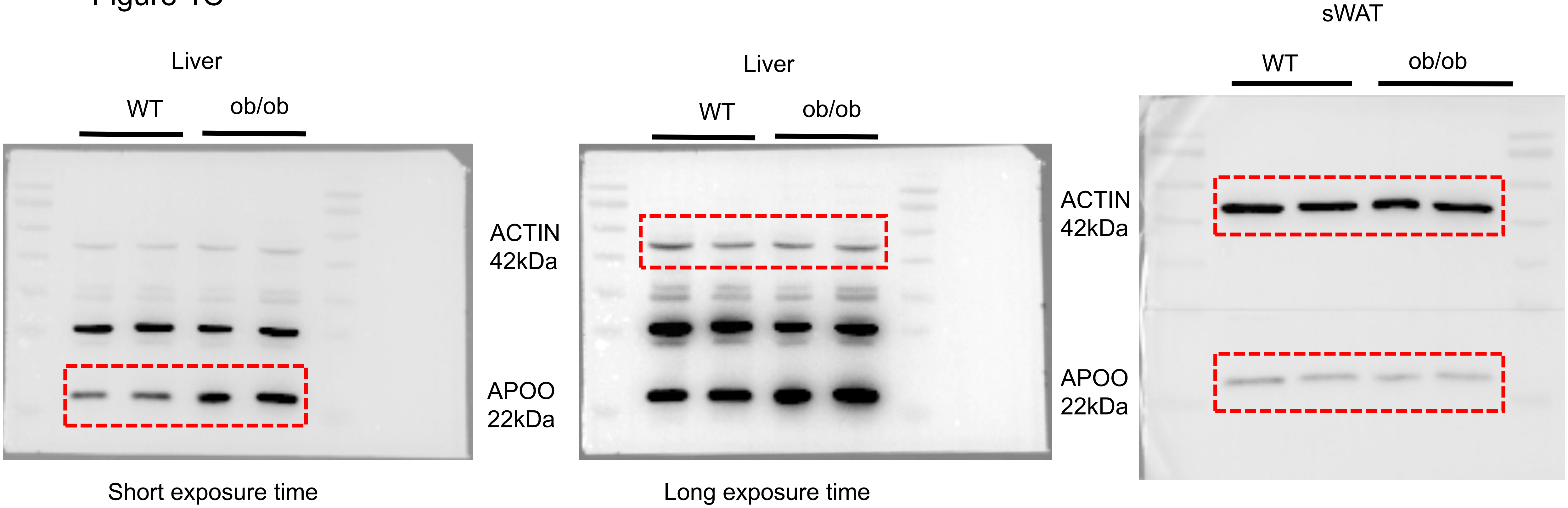

Liver
WT
ob/ob
ACTIN
42kDa
APOO
22kDa
Short exposure time
Liver
WT
ob/ob
ACTIN
42kDa
APOO
22kDa
Long exposure time
sWAT
WT
ob/ob

Figure 1D

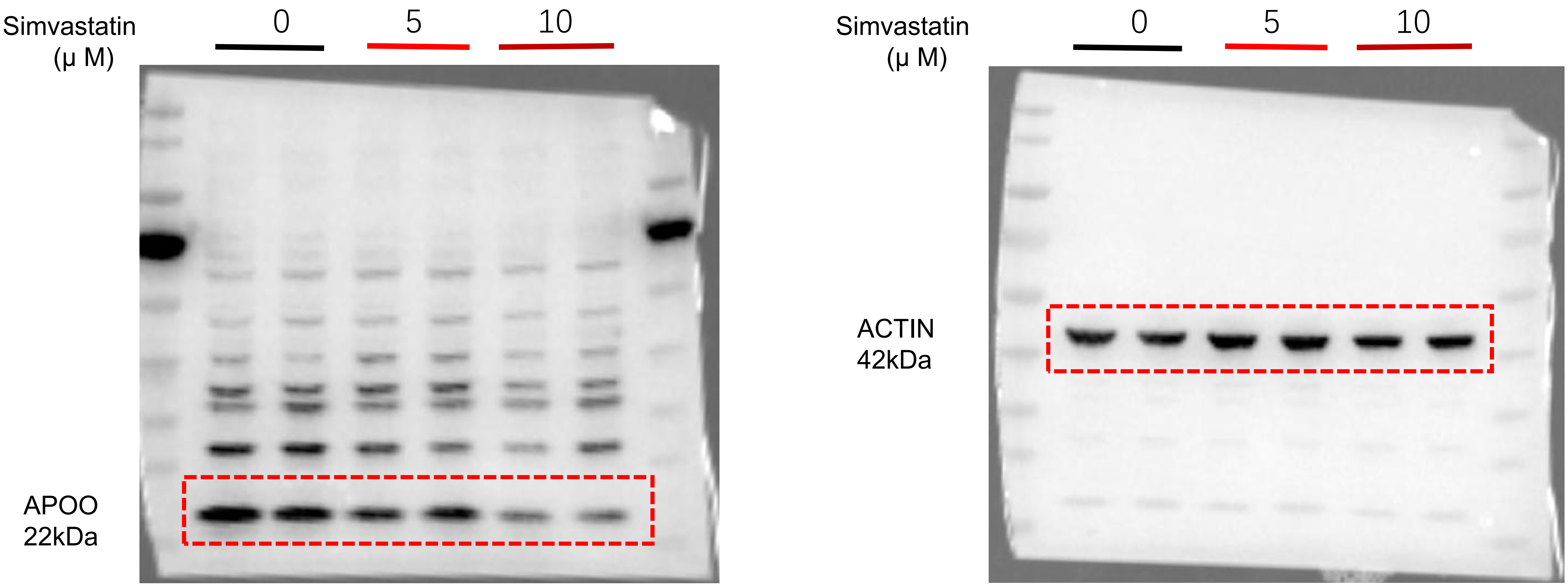
Simvastatin
(μ M)
0
5
10
APOO
22kDa
Simvastatin
(μ M)
0
5
10
ACTIN
42kDa

Figure 1H

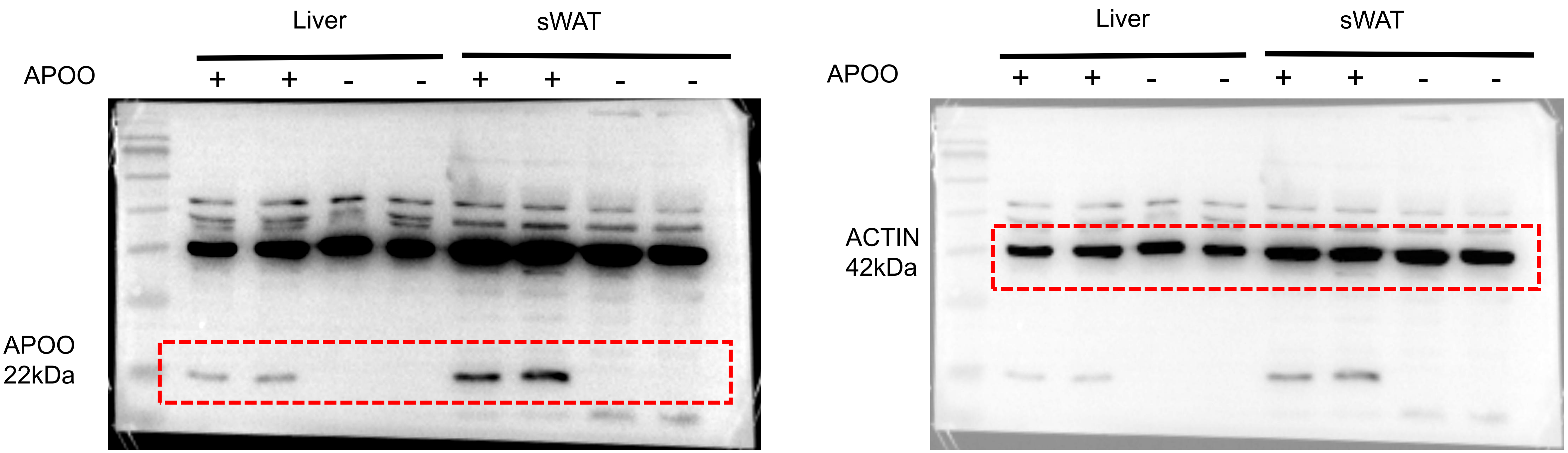

Figure 5H

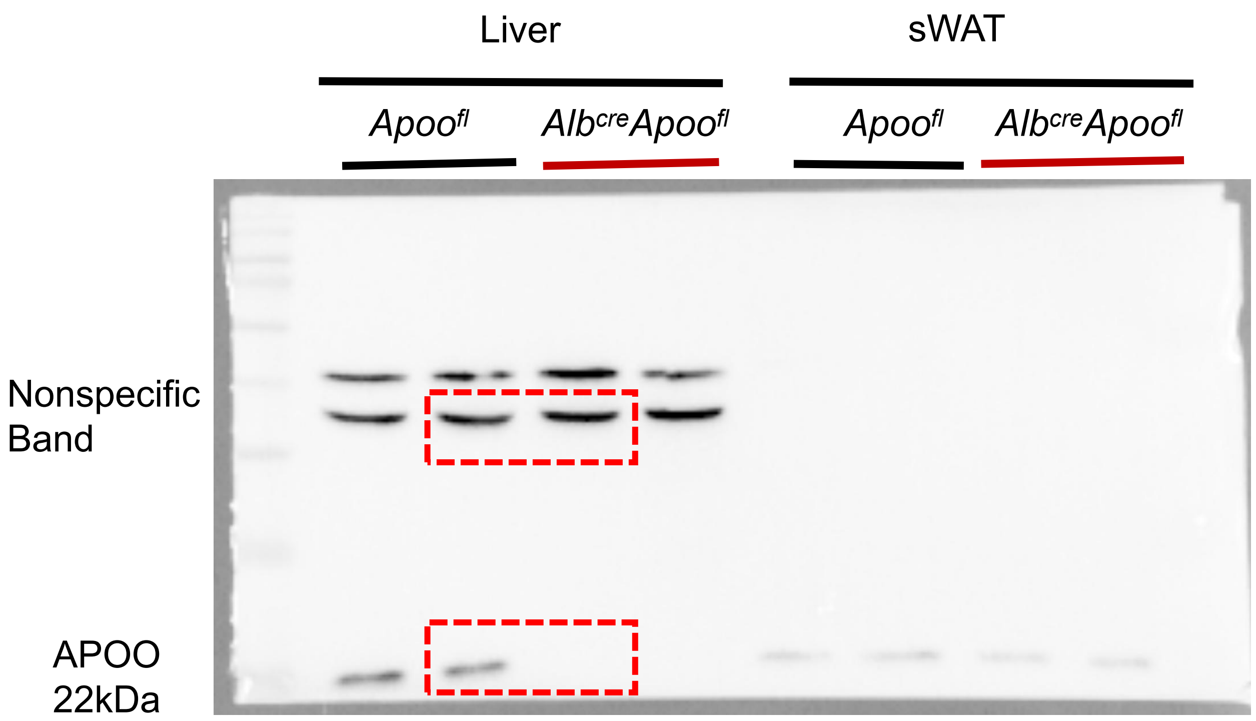
Liver
sWAT
$Apoo^{fl}$
$Alb^{cre}Apoo^{fl}$
$Apoo^{fl}$
$Alb^{cre}Apoo^{fl}$
Nonspecific Band
APOO 22kDa

Figure 7A

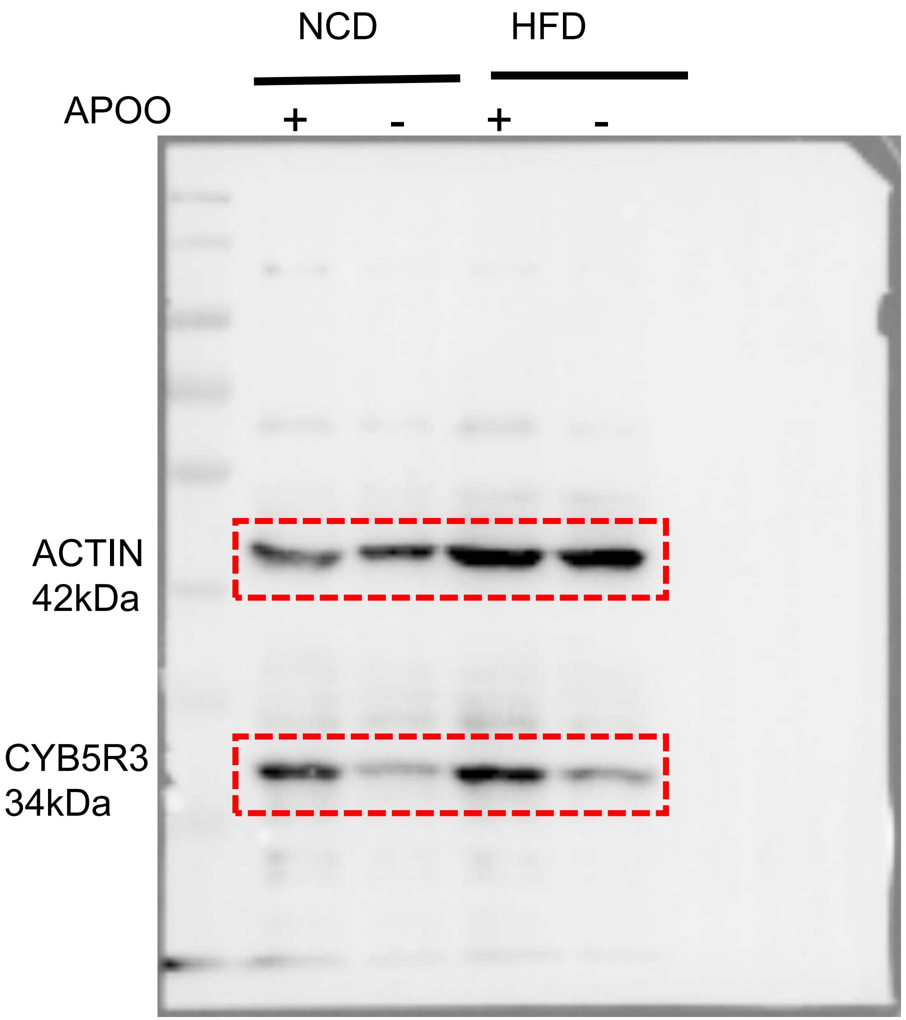

Figure 7D

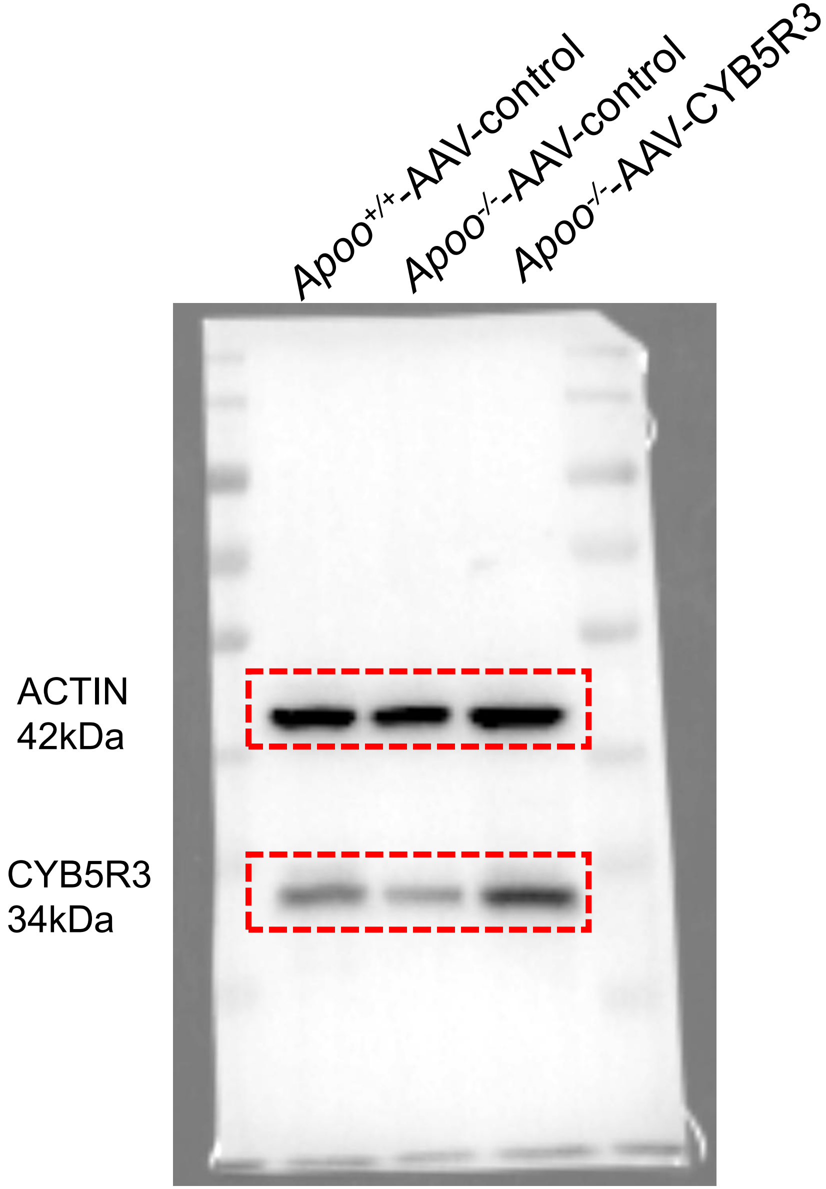

Apoo+/+-AAV-control
Apoo-/--AAV-control
Apoo-/--AAV-CYB5R3
ACTIN
42kDa
CYB5R3
34kDa

Figure 7N

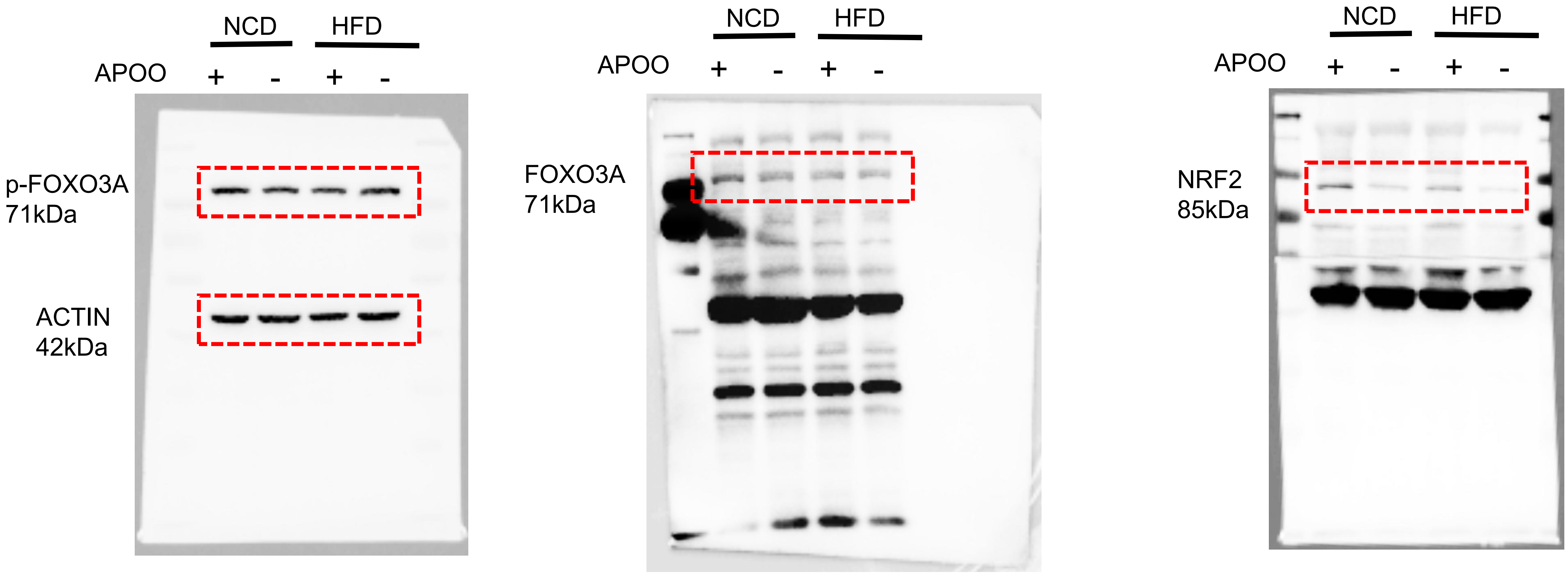

NCD
HFD
APOO
+
-
+
-
p-FOXO3A
71kDa
ACTIN
42kDa
NCD
HFD
APOO
+
-
+
-
FOXO3A
71kDa
NCD
HFD
APOO
+
-
+
-
NRF2
85kDa

Figure 7Q

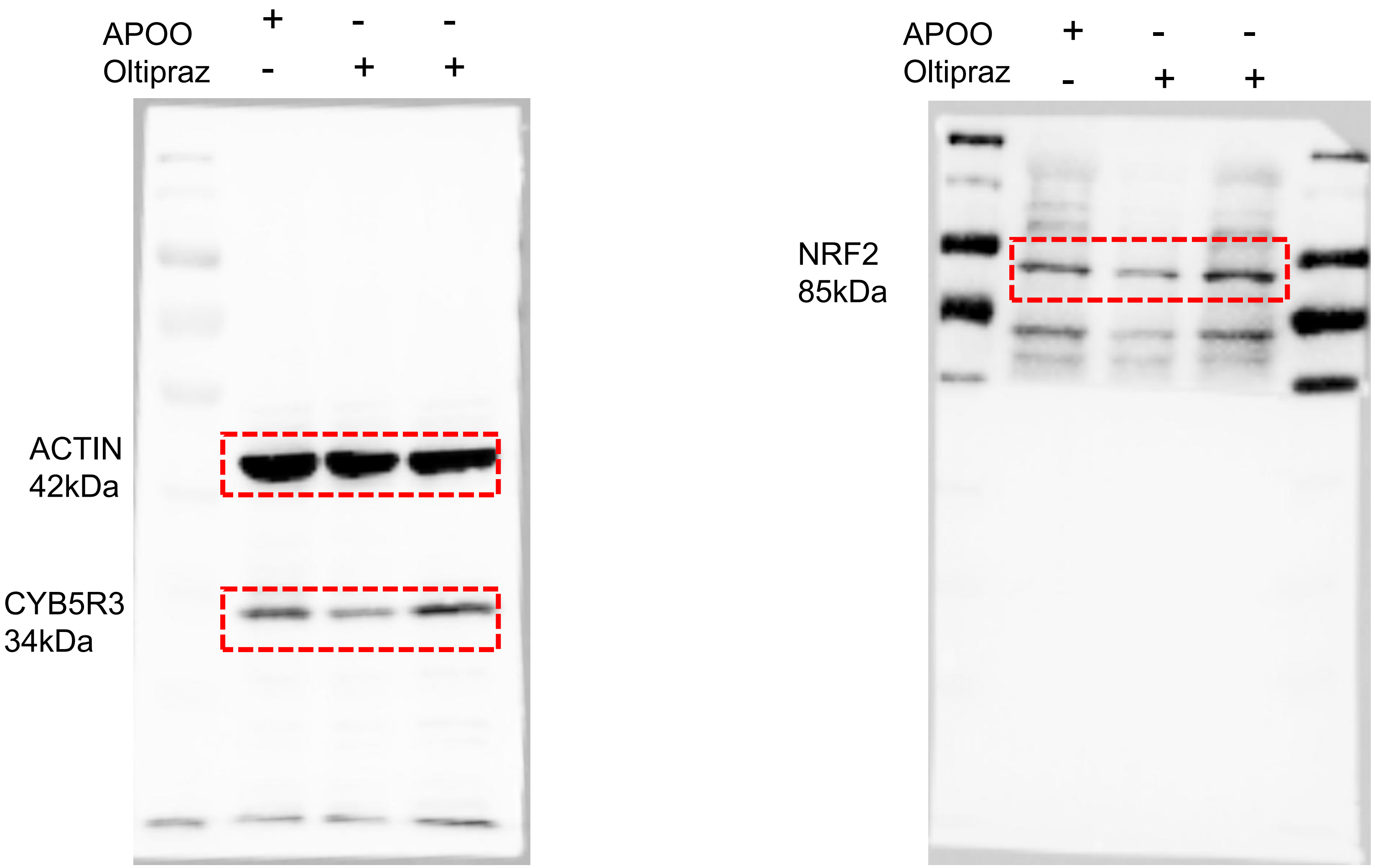

APOO + - -
Oltipraz - + +
ACTIN
42kDa
CYB5R3
34kDa
APOO + - -
Oltipraz - + +
NRF2
85kDa

Supplemental figure 1A

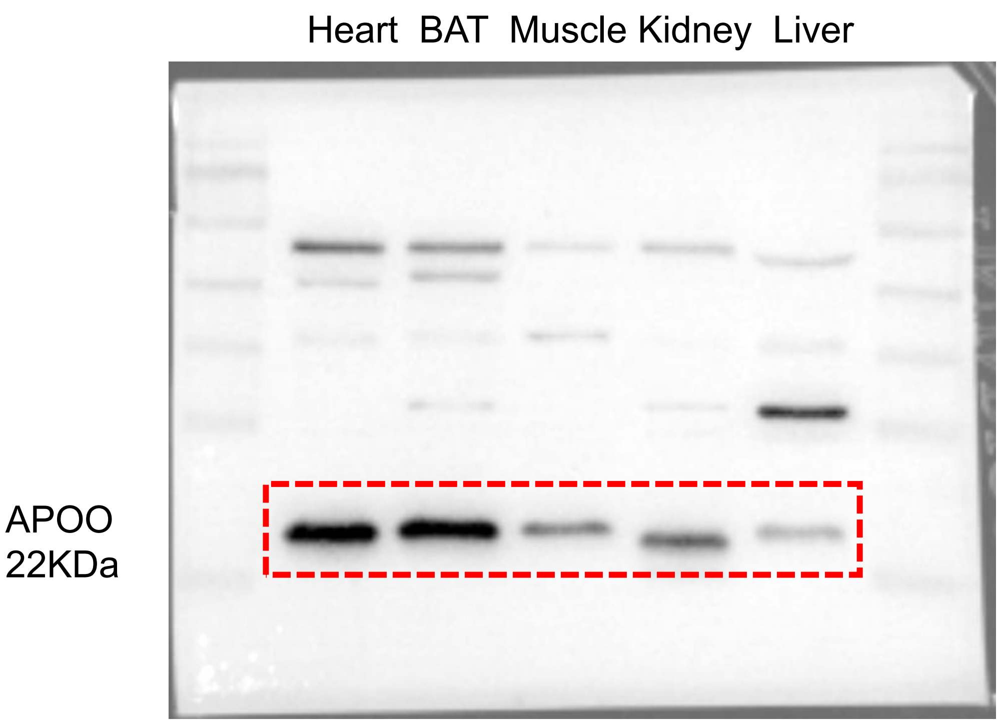

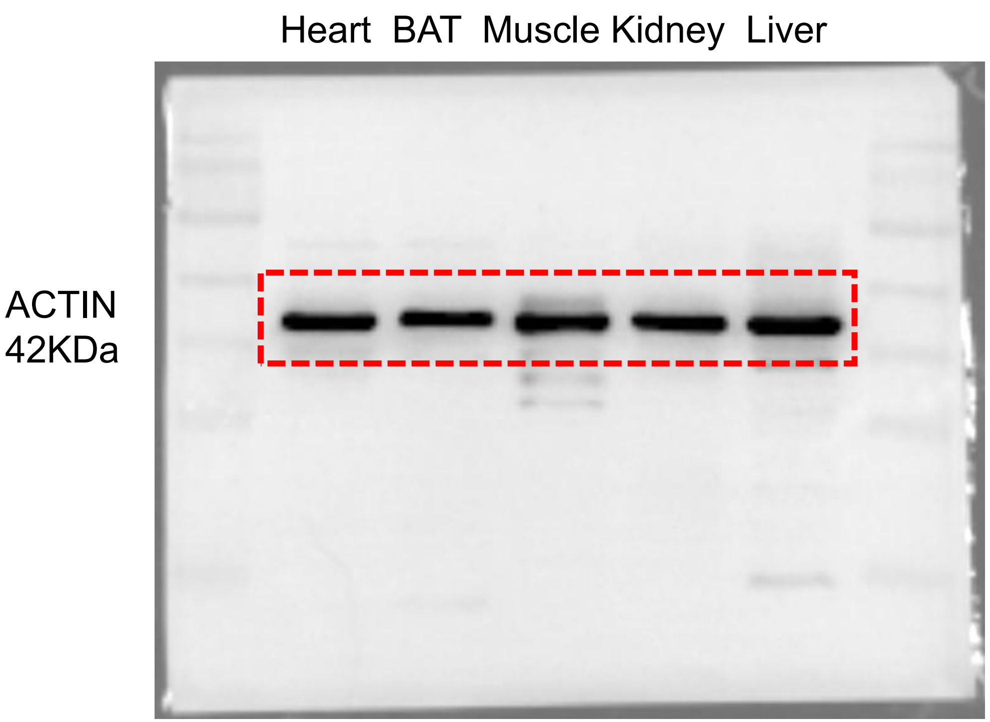

Supplemental figure 1B

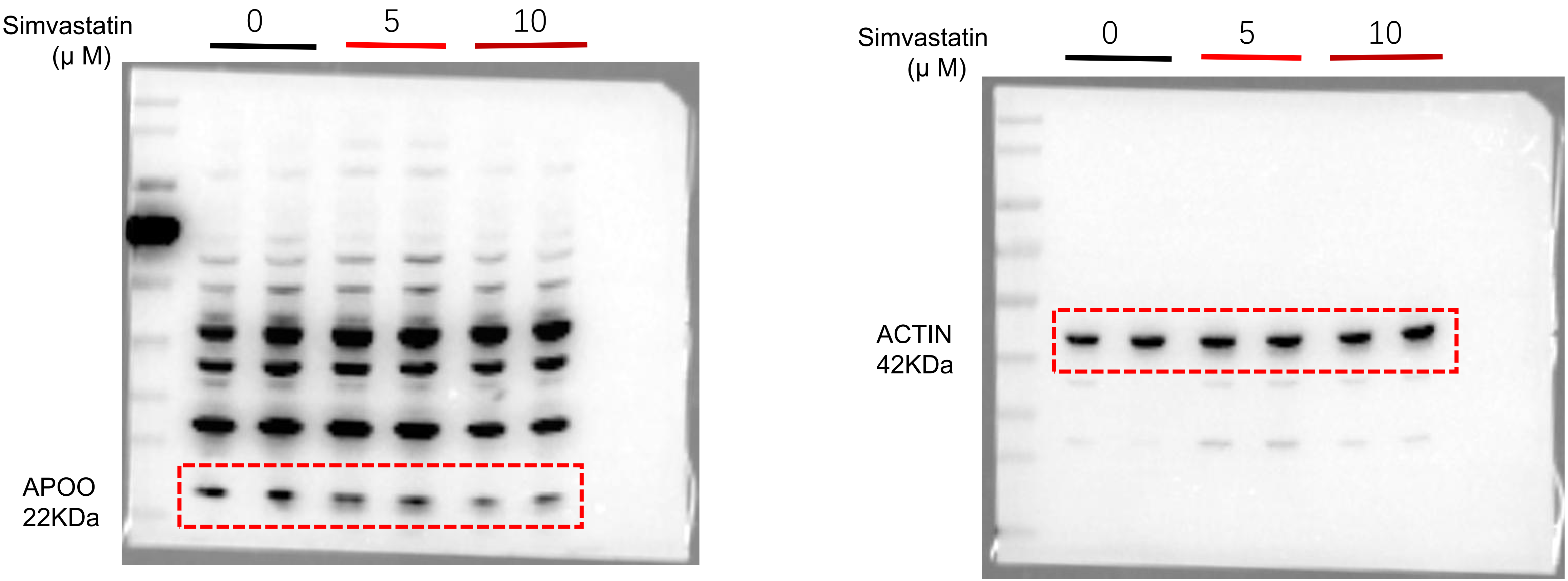

Supplemental figure 2J

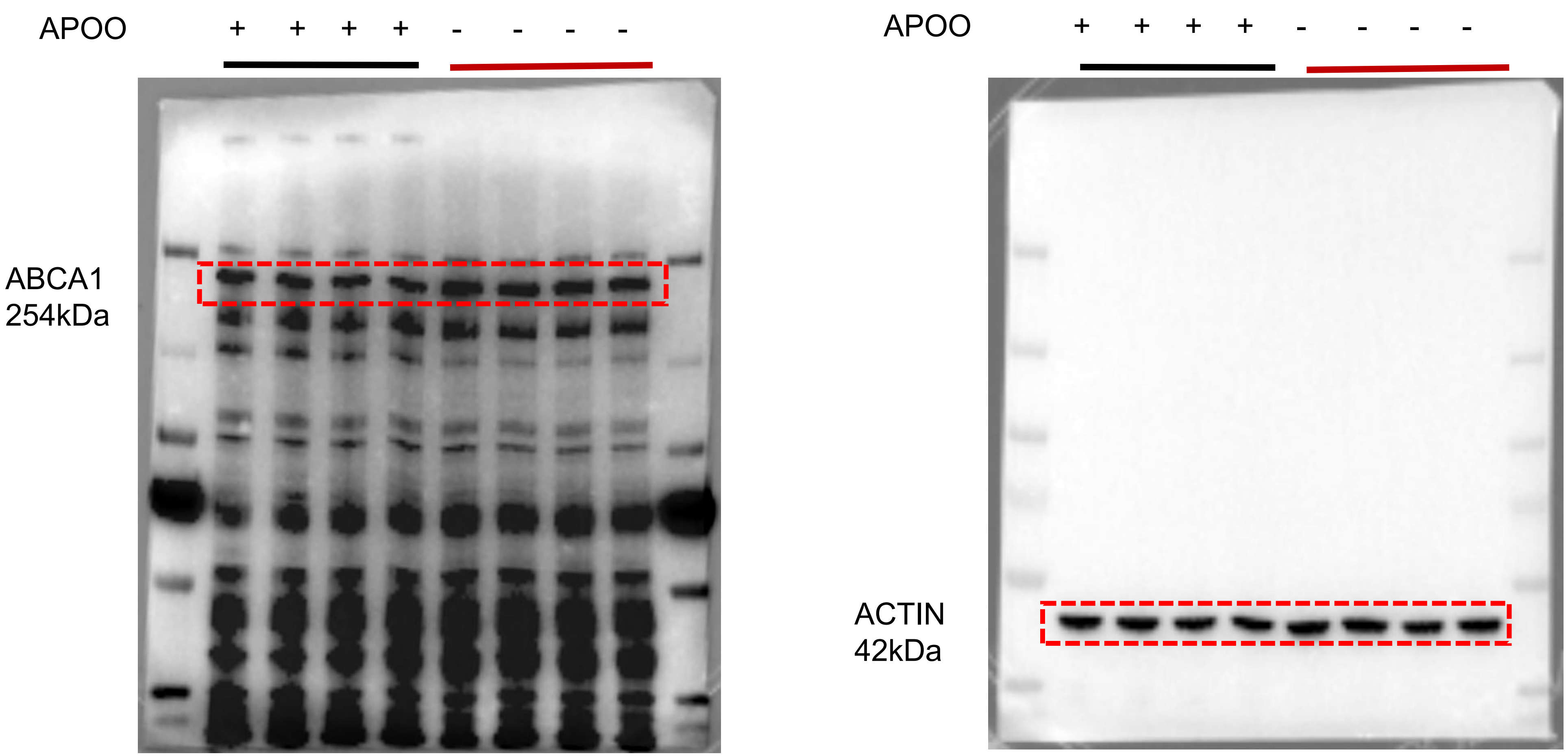

Supplemental figure 2K

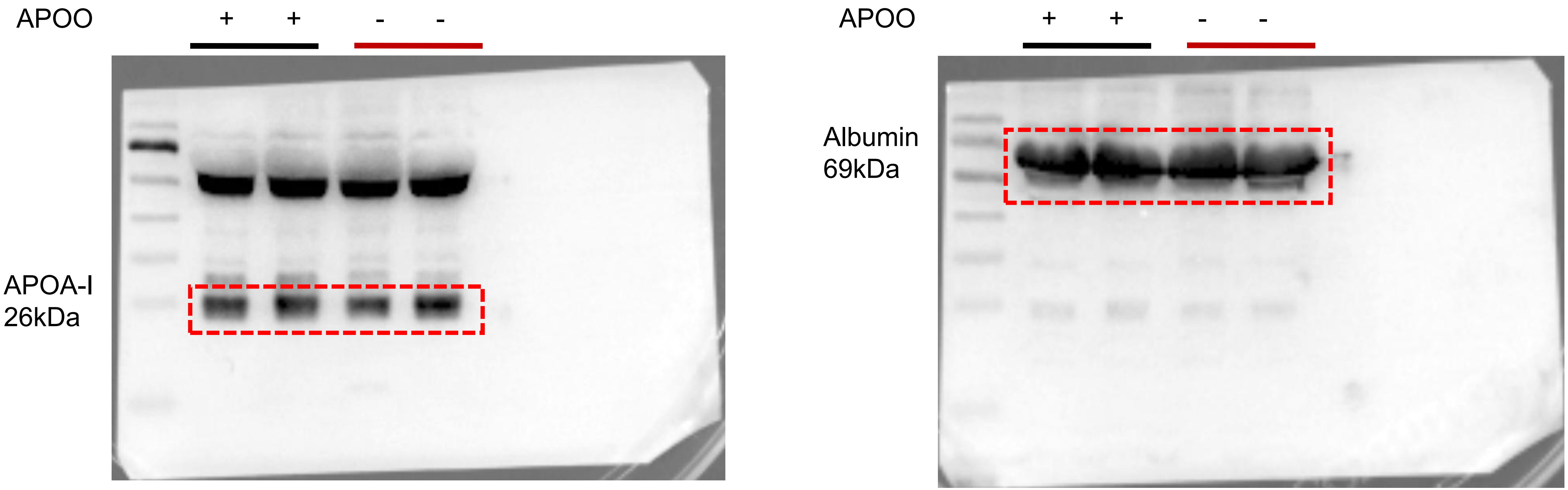

Supplemental figure 4A

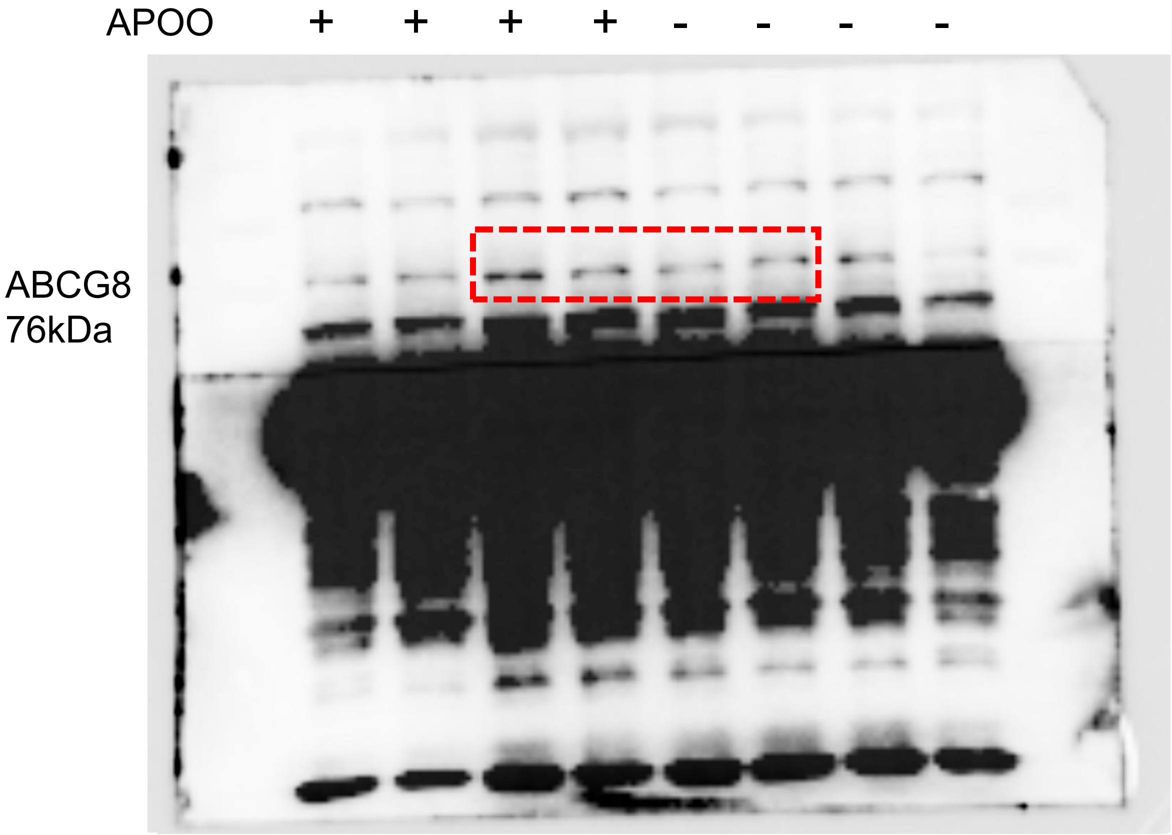

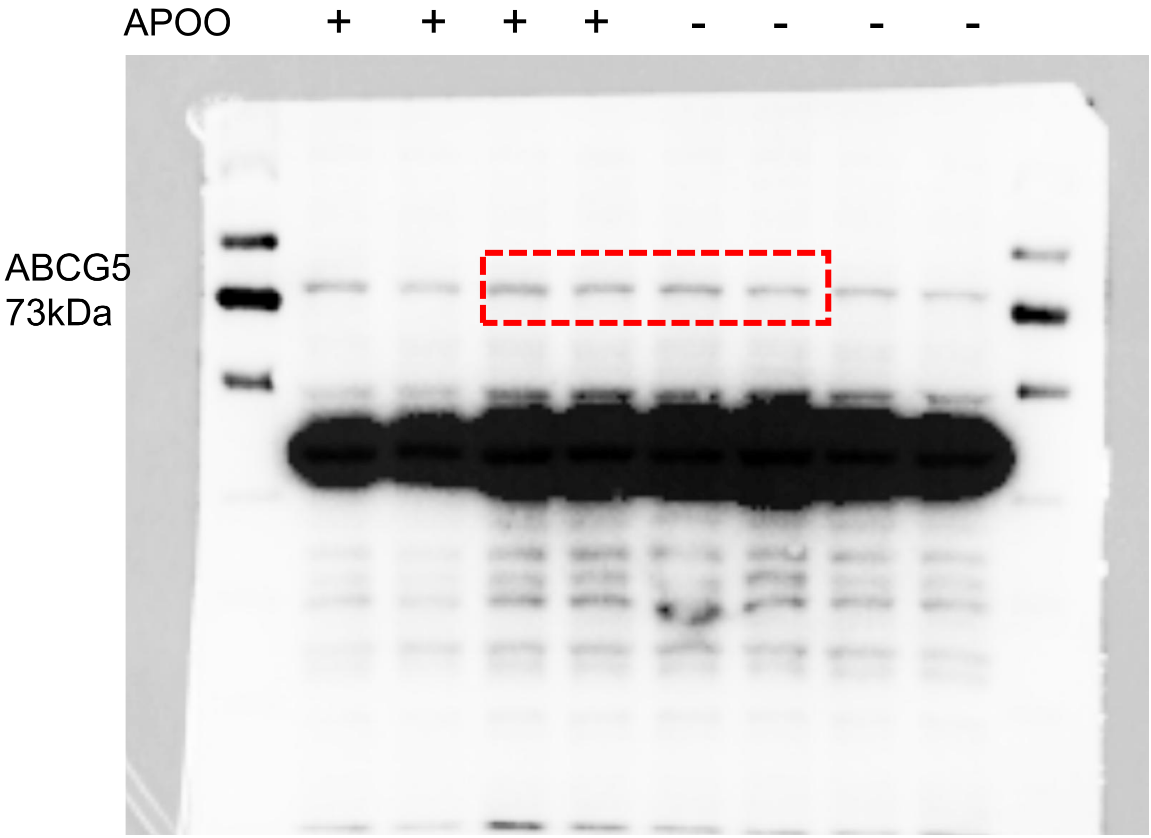

Supplemental figure 4A

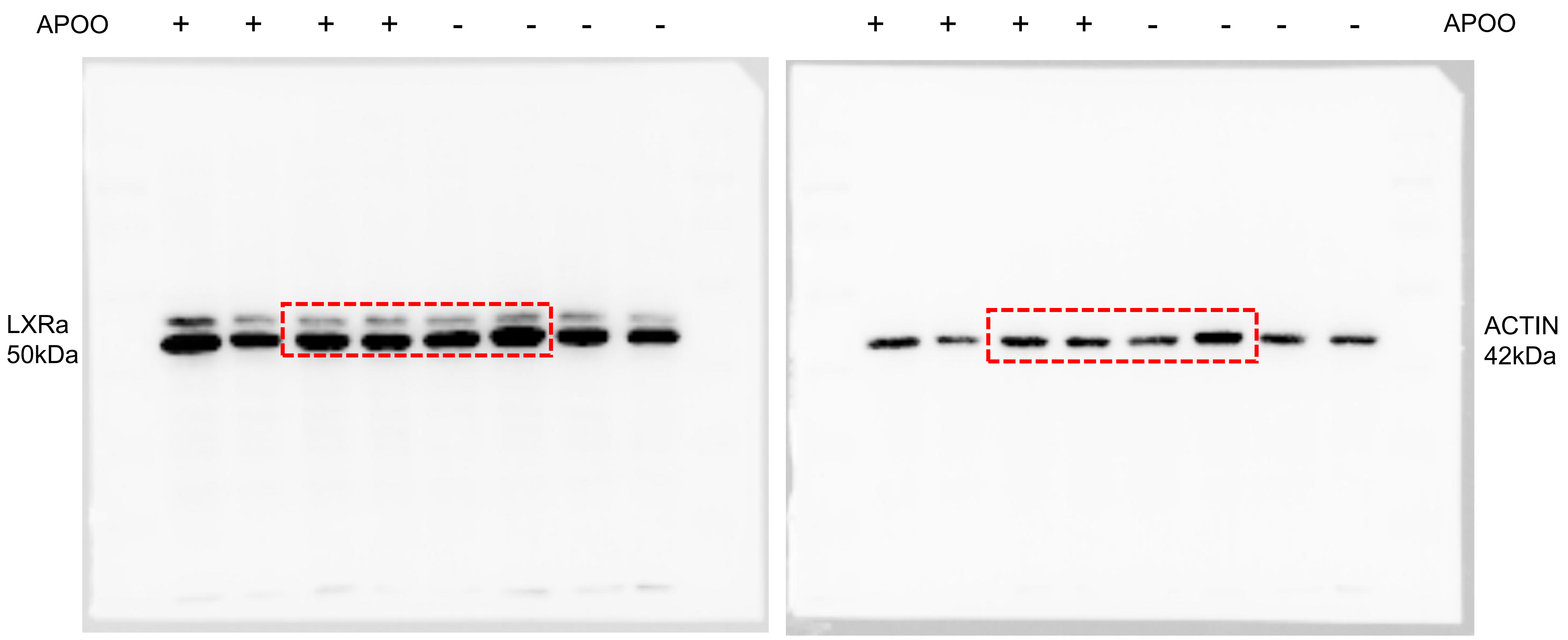

Supplemental figure 5

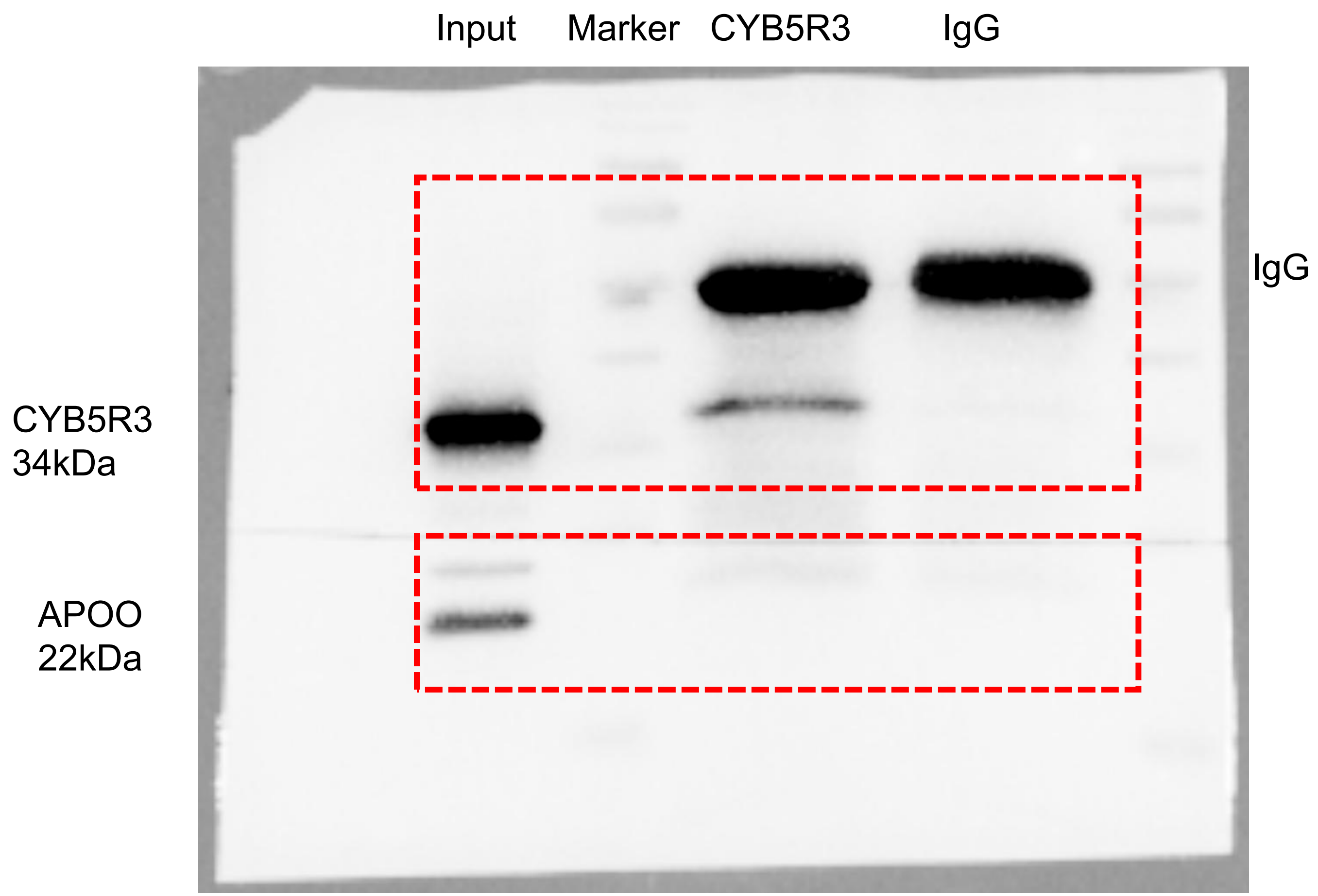

Supplement: Supplementary file 7 — Supplementary Material-Original full length western blots [file 41419_2024_6778_MOESM7_ESM.pdf]
